# Establishing an Evaluation Indicator System for User Satisfaction With Hypertension Management Apps: Combining User-Generated Content and Analytic Hierarchy Process

**DOI:** 10.2196/60773

**Published:** 2024-09-03

**Authors:** Yunfan He, Han Chen, Peng Xiang, Min Zhao, Yingjun Li, Yongcheng Liu, Tong Wang, Jun Liang, Jianbo Lei

**Affiliations:** 1 Center for Health Policy Studies School of Public Health Zhejiang University Hangzhou China; 2 Department of Cardiology Second Affiliated Hospital, College of Medicine Zhejiang University Hangzhou China; 3 Department of AI and IT Second Affiliated Hospital, School of Medicine Zhejiang University Hangzhou China; 4 Intelligent Medical Research Center Zhejiang University Institute of Computer Innovation Technology Hangzhou China; 5 IT Center The First Affiliated Hospital of Xiamen University XiaMen China; 6 Department of Gynecology The First Affiliated Hospital of Xiamen University XiaMen China; 7 School of Public Health Hangzhou Medical College Hangzhou China; 8 Netease Group Hangzhou China; 9 School of Health and Life Sciences University of Health and Rehabilitation Sciences Qingdao China; 10 School of Basic Medical Sciences Shandong University Jinan China; 11 Qingdao Hospital University of Health and Rehabilitation Sciences (Qingdao Municipal Hospital) Qingdao China; 12 National Key Laboratory of Transvascular Implantable Devices Second Affiliated Hospital, School of Medicine Zhejiang University Hangzhou China; 13 School of Medical Technology and Information Engineering Zhejiang Chinese Medical University Hangzhou China; 14 Clinical Research Center Affiliated Hospital of Southwest Medical University Luzhou China; 15 The First Affiliated Hospital Hainan Medical University Haikou China; 16 Center for Medical Informatics Health Science Center Peking University Beijing China

**Keywords:** hypertension management, mobile health, user satisfaction, evaluation indicator system, analytic hierarchy process

## Abstract

**Background:**

Hypertension management apps (HMAs) can be effective in controlling blood pressure, but their actual impact is often suboptimal. Establishing a user satisfaction evaluation indicator system for HMAs can assist app developers in enhancing app design and functionality, while also helping users identify apps that best meet their needs. This approach aims to improve the overall effectiveness of app usage.

**Objective:**

This study aims to systematically collect data on HMAs and their user reviews in the United States and China. It analyzes app usage patterns and functional characteristics, identifies factors influencing user satisfaction from existing research, and develops a satisfaction evaluation indicator system to provide more accurate recommendations for improving user satisfaction.

**Methods:**

We conducted a descriptive statistical analysis to assess the development status of HMAs in both countries and applied the task-technology fit model to evaluate whether the app functionalities align with business needs. We separately summarized the factors influencing user satisfaction in both countries from previous research, utilized the analytic hierarchy process to develop an evaluation indicator system for HMA user satisfaction, and calculated satisfaction levels. Based on these findings, we propose improvements to enhance app functionality and user satisfaction.

**Results:**

In terms of current development status, there were fewer HMAs and user reviews in China compared with the United States. Regarding app functional availability, fewer than 5% (4/91) of the apps achieved a demand fulfillment rate exceeding 80% (8/10). Overall, user satisfaction in both countries was low.

**Conclusions:**

In the United States, user satisfaction was lowest for advertising distribution, data synchronization, and reliability. By contrast, Chinese apps need improvements in cost efficiency and compatibility.

## Introduction

The global prevalence of hypertension is high, with low control rates. Although hypertension management apps (HMAs) have proven effective in controlled settings for helping patients manage blood pressure, user satisfaction and real-world effectiveness are still not well understood. The World Health Organization’s “Global Report on Hypertension: The Race Against a Silent Killer” [1] indicates that the number of patients with hypertension worldwide increased from 648 million in 1990 to 1.278 billion in 2019, with a prevalence rate exceeding 33% and a control rate of only 21%. As a digital health tool [2-4], HMAs can enhance users’ awareness of self-health management [5] and improve drug compliance [6,7], thereby aiding in blood pressure management [8,9]. Clinical trials have confirmed their effectiveness [10-12]. However, before 2015, the number of HMAs was relatively small, with fewer than 10 new apps introduced per year. At that time, users needed more robust core functions to effectively manage their health. Gaps in functional design led to poor user satisfaction [13] and limited effectiveness [14,15]. Over 80% of mobile health app users exhibited abandonment behavior [16], with 30% of mobile health apps being abandoned within a month [17], and only 6.6% of users continuing to use HMAs long-term [18].

Understanding the current status and functional characteristics of HMAs and developing a user satisfaction evaluation indicator system (USEIS) can significantly improve HMA design, leading to enhanced user satisfaction and effectiveness. By analyzing user demand and app functionality, developers can identify unmet needs and refine app features accordingly. Comparing USEISs enables targeted improvements on factors with high importance but low user satisfaction. By understanding the product characteristics and overall user satisfaction of various HMAs through the evaluation indicator system, users can select the app that best meets their needs.

Both the United States and China, which face the most severe cases of hypertension worldwide [19,20], have launched numerous HMAs [21,22]. This indicates strong market prospects for HMAs in both countries. Comparing and analyzing the product features and user satisfaction of HMAs are crucial for optimizing their functionality and performance in both markets. Analyzing app demand and functionality in both countries and constructing a USEIS based on previous research [23] are crucial for accurately evaluating and improving user satisfaction.

Current research on HMA user satisfaction can be broadly divided into offline user research and online user data analysis. Most studies in this area concentrate on user satisfaction surveys and the factors influencing them. Offline user research typically involves methods such as interviews [24], questionnaire surveys [25-27], literature reviews [28], and case analyses [29]. For instance, studies such as those by Ramirez et al [30] and Breil et al [31] provide insights into user experiences through these traditional research approaches. Online user data analysis uses techniques such as manual coding [32,33] and text mining [34,35] to extract information about HMAs’ main functions from app descriptions and user reviews and to investigate factors influencing user satisfaction (eg, Alnooh et al [36] and He et al [23]). Additionally, Haggag et al [37] utilized user-generated content to preliminarily explore user satisfaction and influencing factors of mobile health apps. Desmal et al [38] and Taylor et al [39] established satisfaction evaluation indicator systems for mobile government portal websites and consumer health information websites through literature reviews and assessed satisfaction through case analyses. Meanwhile, other scholars have developed user satisfaction indicator evaluation systems for products and services across various fields using expert Delphi [40,41] and analytic hierarchy process (AHP) [42,43], a decision analysis method for solving complex decision problems. The process of users posting ratings and reviews is a direct expression of their satisfaction with the app’s functionality and services. This user-generated content can reveal the main attributes and genuine emotional tendencies that users focus on while using the app in real time. By analyzing this content, it is possible to gain an accurate understanding of users’ health management needs and develop more comprehensive app features to better support users in managing their own health.

However, existing studies have not sufficiently analyzed HMA demands and functions nor established a USEIS based on influencing factors. These studies have primarily summarized HMA functions through manual coding or content analysis [44-46] and have categorized the distribution of app functions. However, they have not assessed the apps’ functional availability—the extent to which their functionality meets business needs, specifically the self-management demands for hypertension as outlined in clinical guidelines [22]. This field lacks quantitative research driven by real-world big data [47,48] and evaluation research on app user satisfaction [49-51]. Although we previously used text mining to extract the main factors influencing HMA user satisfaction in the United States and China [23], we did not explore the importance of these factors or develop a USEIS.

Therefore, this study comprises 2 main parts: functional availability analysis and user satisfaction analysis of HMAs in China and the United States. First, we summarize the main functions and business demands of HMAs in both countries. Using the theory of task-technology fit (TTF; an information technology evaluation method based on task and technology matching), we calculate the compatibility between functions and demands to analyze functional availability and explore how well these HMAs meet user needs. Functional availability analysis helps understand the development status of HMAs and quickly identifies functions that have not yet met user needs. Second, based on the influencing factors of user satisfaction identified in previous studies [23], we determine the specific indicators and their weights affecting user satisfaction in the United States and China using the AHP. User satisfaction analysis helps identify the core elements affecting HMA usage and assists developers in crafting precise optimization strategies. For research significance, this study not only offers a suitable method and process for analyzing functional availability and user satisfaction of mobile health apps but also uncovers the differences in functional availability and user satisfaction between Chinese and American HMAs. It provides suggestions for enhancing the quality of HMAs.

## Methods

### Theoretical Basis and Methodological Process

**Figure 1:**
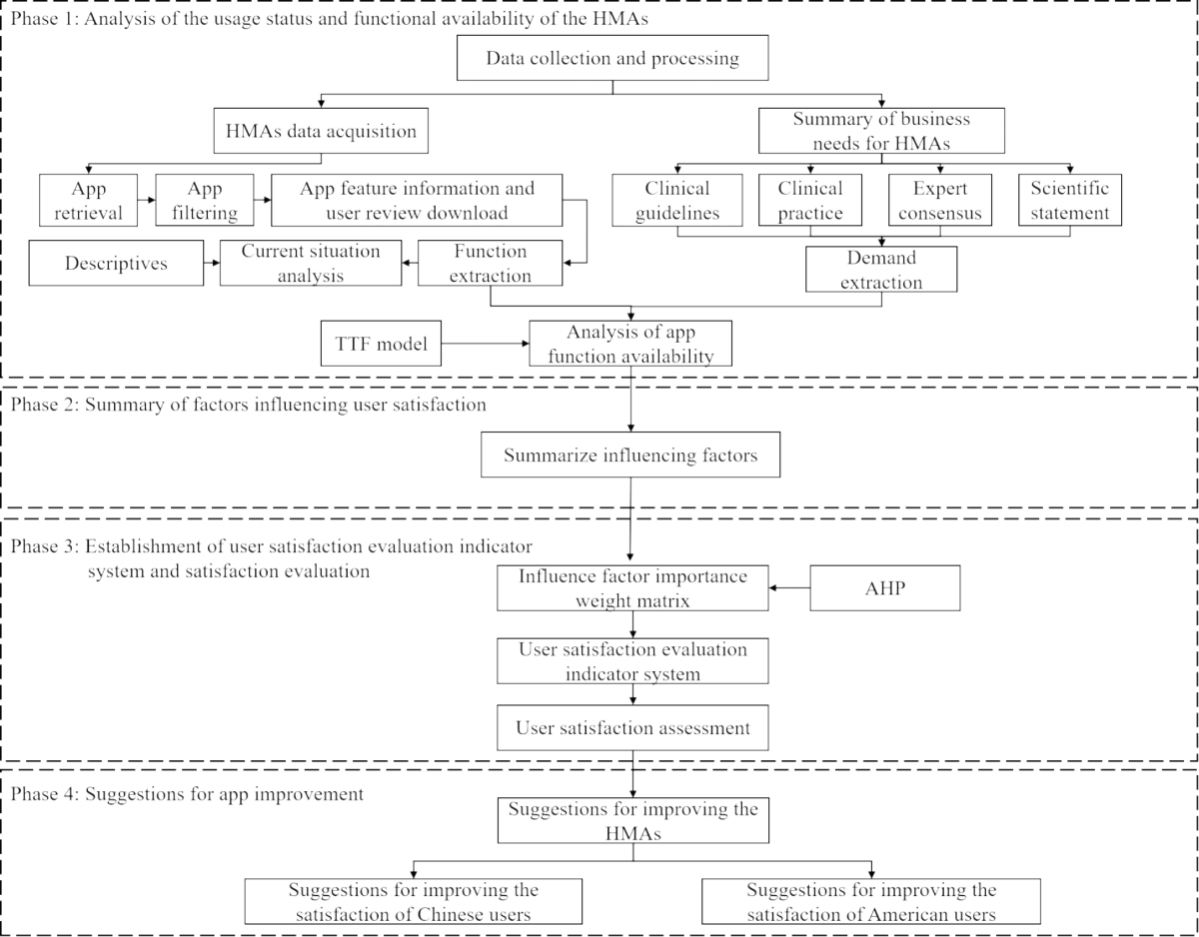
Method flowchart. AHP: analytic hierarchy process; HMA: hypertension management app; TTF: task-technology fit.

This article, based on TTF theory and decision theory, uses descriptive statistics, the TTF model, and AHP to evaluate the functional availability of HMAs in China and the United States. It extracts factors affecting user satisfaction from user reviews and establishes a USEIS. The study compares the differences in results between the 2 countries. The methodology is divided into 4 main parts: analysis of app usage status and functional availability, induction of factors affecting user satisfaction, establishment of a USEIS, and suggestions for app improvement. The detailed method flow is illustrated in Figure 1.

### Data Acquisition, Filtering, and Preprocessing

**Figure 2:**
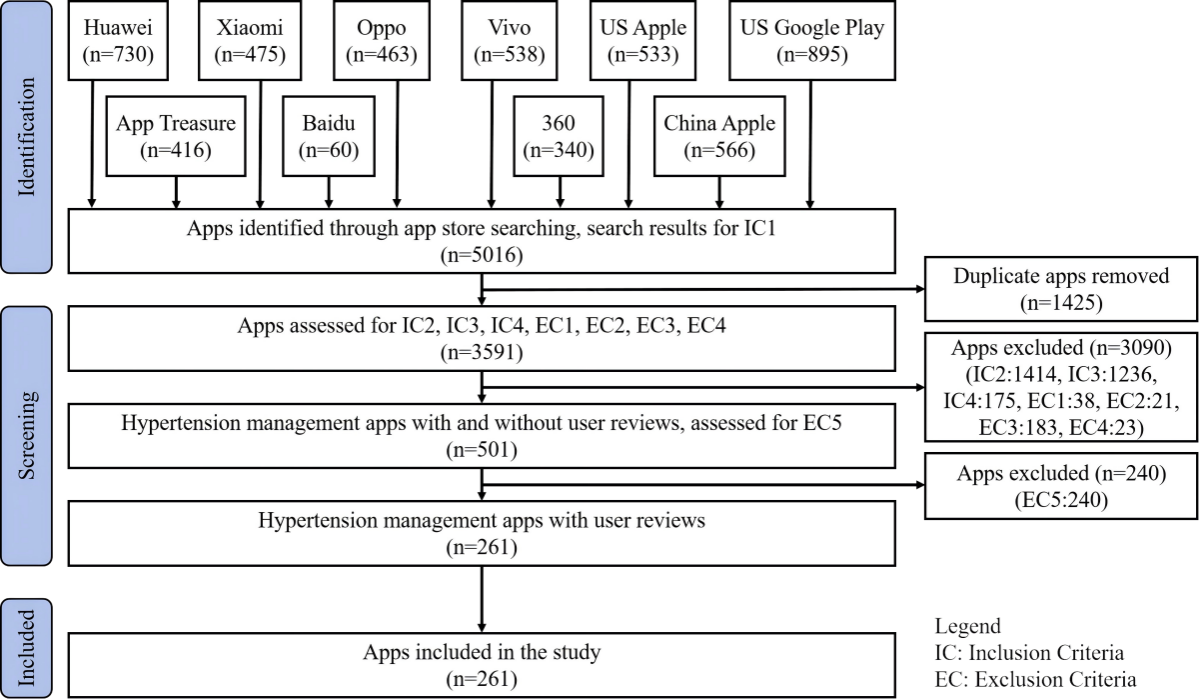
Flowchart of the hypertension management app screening process.

We used HMA user reviews from the United States and China, obtained in a previous study [23]. This includes a brief description of the process for obtaining and screening HMAs, as well as preprocessing user reviews from both countries. In April 2023, we retrieved 5016 related apps using 2 English keywords across 2 American app stores and 10 Chinese keywords across 8 Chinese app stores. Following deduplication, 3591 apps remained (for detailed search terms and results, see Table S1 in Multimedia Appendix 1). Two researchers (YFH and JL) independently conducted initial screenings, with a third researcher (HC) arbitrating to identify HMAs, resulting in 261 HMAs with user reviews. Of these, 170 apps were exclusive to app stores in the United States, 41 to app stores in China, and 50 apps were available in both (for detailed inclusion and exclusion criteria and screening flowcharts, see Table S2 in Multimedia Appendix 1 and Figure 2, respectively (also see Figure S1 in Multimedia Appendix 1). To gather and preprocess app user reviews, we used the Qimai Mobile Application Data Analysis Platform [52] and self-developed Python (Python Foundation) scripts, accumulating a total of 295,927 user reviews and rating data from both the United States and China, including 250,193 US reviews and 45,734 Chinese reviews. To ensure the authenticity and reliability of these reviews, we performed data preprocessing. This involved removing reviews from robot accounts (identified and deleted using the tweetbotnot package [53]), duplicates, invalid reviews (including garbled characters, emoticons, non-Chinese or non-English content, and meaningless reviews such as advertisements), reviews with mismatched emotional polarity and ratings [54], and blank reviews. Ultimately, this study analyzed 116,686 US and Chinese user reviews to develop a USEIS (for the number of apps and user reviews, see Table S3 in Multimedia Appendix 1; for details on data acquisition, screening, and preprocessing processes, see the “Obtaining, Screening, and Preprocessing of Hypertension Management Apps and Their User Reviews” section in Multimedia Appendix 1).

### Extraction and Validation of App Product Features

#### App Product Information Extraction

To study the current trends, usage, and technological attributes of HMAs in the United States and China, this study builds upon previous research [46,55] to develop a comprehensive app information extraction framework. This framework extracts the product features of each HMA, and we used descriptive statistical methods to analyze the distribution of these features. The framework encompasses 5 dimensions: basic information, listing status, user usage, development standardization, and technical completeness. A detailed description of the framework is presented in Table S4 in Multimedia Appendix 1. To ensure consistency in information extraction, 2 researchers (YFH and JL) extracted all product features of the 261 HMAs, with any inconsistencies resolved by a third researcher (HC). Additionally, to assess the consistency of the data extraction between the 2 researchers, we randomly selected 1 field from the 20 extracted fields and calculated the consistency. The kappa consistency coefficient was 0.89, indicating a high level of agreement between the researchers.

#### App Feature Information Extraction

This study, building upon previous research [22], involved manually extracting and summarizing the functional characteristics of HMAs in the United States and China based on app store descriptions using the induction method. First, comprehensive function descriptions for 261 HMAs—including blood pressure measurement, blood pressure recording, and doctor-patient communication—were manually extracted from app store descriptions, software interface screenshots, and function module introductions provided in the app stores. These descriptions were then consolidated, standardizing various terminologies for identical functions and categorizing them into distinct functional dimensions based on similarity. This process resulted in a standardized functional summary table (Table S5 in Multimedia Appendix 1), which helped ensure consistency in functional descriptions across all apps. Finally, we separately summarized the specific functions of HMAs in both countries.

### Analysis of App Function Availability Based on the Task-Technology Fit Model

#### Overview

The availability of app functionality is a crucial metric for evaluating its completeness, specifically assessing whether the app provides sufficient features to meet users’ needs. This concept pertains to how well an app’s existing functions address business demands [22]. The TTF model is an information technology evaluation model that quantifies how well information technology can perform specific tasks for users by assessing the compatibility between information technology functions and task demands. The model suggests that if the alignment between technical functions and task demands is high, technology can enhance task performance. This study drew inspiration from the TTF concept in the TTF model to evaluate the functional availability of HMAs by analyzing the alignment between their functions and the needs of hypertension management tasks. It assessed how well these apps promote users’ ability to complete hypertension management tasks. The study systematically reviewed the main functions of HMAs in the United States and China, using the TTF model to calculate the adaptation of specific functions and business demands in each country separately, to evaluate their functional availability. Additionally, the study aimed to understand the apps’ effectiveness in helping users in both countries manage their hypertension.

#### Summary of Business Demands

First, we compiled and reviewed the latest clinical guidance documents on hypertension management and diagnosis from the official websites of hypertension institutions in both countries, as well as from international institutions and English and Chinese literature databases. This review was supplemented by consultations with expert clinicians. Finally, a clinical expert in the cardiovascular field systematically reviewed the clinical guidance documents for hypertension in the United States and China and summarized the business needs of users in both countries for independently managing hypertension. For details on the process of summarizing app business demands, see the “Summary of App Business Requirements” section in Multimedia Appendix 1.

#### Functional Availability Analysis of the Apps

For overall app functionality availability, we will match all the functions of HMAs in each country with the business needs for hypertension self-management specific to that country. This will help identify any business needs that lack functional support, highlighting areas where the apps have not yet provided adequate functionality. For individual app functional availability, we matched the specific functions of each HMA in each country with the business needs for hypertension management in that country. Based on these matching results, we created function demand adaptation maps for HMAs in the United States and China. We then calculated the average functional utilization rate (see Equation 1) and demand satisfaction rate (see Equation 2) for apps in both countries, analyzing the accuracy and completeness of the app’s functional design. The app functional utilization rate refers to the extent to which app functions meet business needs. The app demand satisfaction rate indicates the proportion of hypertension management needs that have been met. Based on the matching results of each app’s functions and demands, we summarized the main unmet needs for HMAs in both countries. Finally, we compared the functional differences between HMAs in the United States and China, analyzing the specific functions and functional availability in each country.

App functional utilization rate = (Number of functions that meet the demands)/(Total number of app functions) **(1)**

App demand satisfaction rate = (The number of demands that the app has met)/(Total number of demands) **(2)**

### Constructing a User Satisfaction Evaluation Indicator System

#### System Design

Based on the factors influencing user satisfaction with HMAs in the United States and China identified in a previous study [23], we further used AHP to construct a USEIS. AHP is a decision-making method that establishes a hierarchical structure to convert subjective judgments into pairwise comparisons of the importance of various factors. We innovated the construction of the weight matrix by quantifying the proportion of user reviews to enhance objectivity and reliability in the decision results. Additionally, we referred to multiple relevant studies [56-59] to ensure the reliability of the evaluation indicator system construction process.

#### Building an Evaluation Indicator System Framework

Based on the AHP decision path model of “target layer-criterion layer-indicator layer,” this study used significant influencing factors from previous research [23] to summarize the evaluation indicator system framework for HMA user satisfaction in the United States and China. First, a framework for evaluating user satisfaction in both countries was constructed according to the decision-path model. Second, factors significantly influencing satisfaction among Chinese and American HMA users were used as specific indicators in the indicator layer of the framework. These indicators were then classified into criteria based on attribute similarities. The indicators, indicator weights, and criterion weights for China and the United States are independent and distinct. These dimensions were then used as specific criteria for the criterion layer. Finally, user satisfaction was defined as the specific goal of the target layer.

#### Determining Indicator Weights

This study followed the analytical process of AHP [60] to determine the weights of each layer of indicators in the 2 evaluation indicator system frameworks. First, using the clustering results from the latent Dirichlet allocation topic model in the previous study [23], the frequency proportion of corresponding reviews for each topic from the United States and China was used as the basis for each indicator. The frequency proportions of each criterion were calculated by summing the frequency proportions of the indicators within the same criterion layer. Next, the importance scale correspondence table was created based on the maximum proportion difference between different indicators and criteria within the same evaluation indicator system, and the scale of levels 1-9 in the indicator and criterion judgment matrix. Hierarchical single sorting and consistency tests were then performed. Based on the importance scale table and the proportional differences between various criteria and indicators, a corresponding judgment matrix was constructed. This matrix was used to calculate the eigenvalues and eigenvectors through hierarchical single sorting. Eigenvectors corresponding to the maximum eigenvalue were identified, and a consistency coefficient verification was performed. A consistency coefficient of ≤0.1 indicates that the feature matrix is consistent and that the normalized maximum eigenvector can be used as the weight for the corresponding indicator [61]. Finally, hierarchical total ranking and consistency testing were conducted. Using the standardized feature vectors that passed consistency testing as the importance weights for the corresponding indicator and criterion layers, we calculated the comprehensive weights of each indicator in the indicator layer relative to the target layer through a linear weighted sum. Combined consistency testing and overall consistency testing were then performed. If both the combined and overall consistency coefficients are less than or equal to 0.1, consistency is present [61]. The entire indicator weight calculation was performed using a self-developed Python (Python Foundation) script.

#### User Satisfaction Assessment

We calculated the overall user satisfaction and satisfaction with each evaluation indicator for HMAs in the United States and China based on the 2 established evaluation indicator systems (which were constructed based on user reviews in this study). First, this study is based on both positive (satisfied) and negative (unsatisfied) deviations in user reviews from the 2 countries, as identified in the previous study [23]. The proportion of user reviews with a satisfied attitude toward a particular topic was used as the initial evaluation value of user satisfaction for that topic. Then, based on the indicator weights from the USEISs in the United States and China and the satisfaction levels for each indicator, we calculated the satisfaction for each criterion and the overall user satisfaction using a linear weighted sum method.

### Ethical Considerations

All data in this study are sourced from internet mobile app stores, and all app information and user reviews can be publicly accessed. Therefore, this study does not involve any medical ethics issues.

## Results

### Development and Use Status of the App

#### Trend of Changes in the Number of Apps and the Frequency of Version Updates

**Figure 3:**
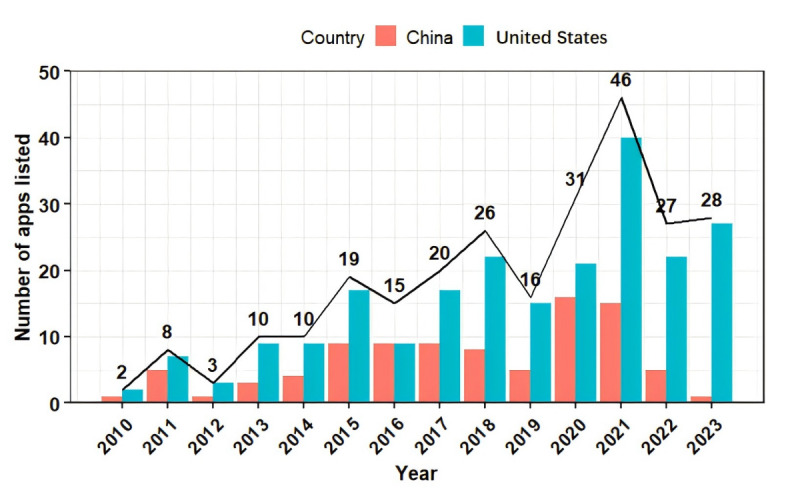
The trend in the number of hypertension management apps.

For app development and listing, the 261 HMAs were first introduced in 2010. From 2010 to 2023, the annual number of listed HMAs generally exhibited a stepwise upward trend, with an average annual growth rate of 51.48% (6.692/13). The number peaked in 2021 and has decreased slightly since then. The number of HMAs listed in Chinese app stores each year was relatively small, often not exceeding 10, while the number listed in American app stores showed a fluctuating upward trend, significantly surpassing that of Chinese app stores (Figure 3). It is important to note that because the same app may be listed in both US and Chinese app stores, the total number of HMAs listed each year does not equal the sum of the number of apps listed in each store.

**Figure 4:**
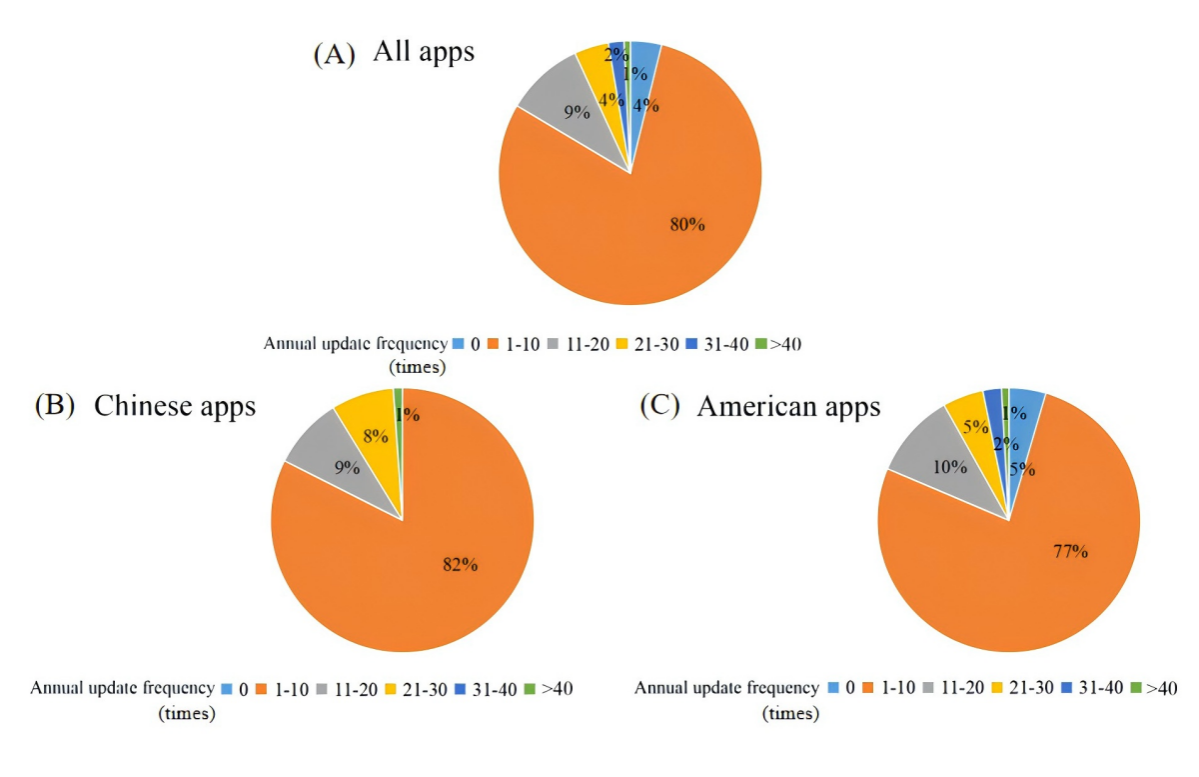
The proportion of annual average update frequency of hypertension management apps.

For app version updates, the 261 HMAs underwent a total of 5508 version iterations, averaging 21 updates per app. Each app was updated approximately 6 times a year. Among these, apps listed in Chinese app stores were updated 2719 times (an average of 29 updates per app), with each app being updated about 7 times a year. By contrast, apps in US app stores were updated 4870 times (an average of 22 updates per app), also with an average of 7 updates per year. The annual update frequency of apps is illustrated in Figure 4.

#### Product Feature Distribution of Apps

**Figure 5:**
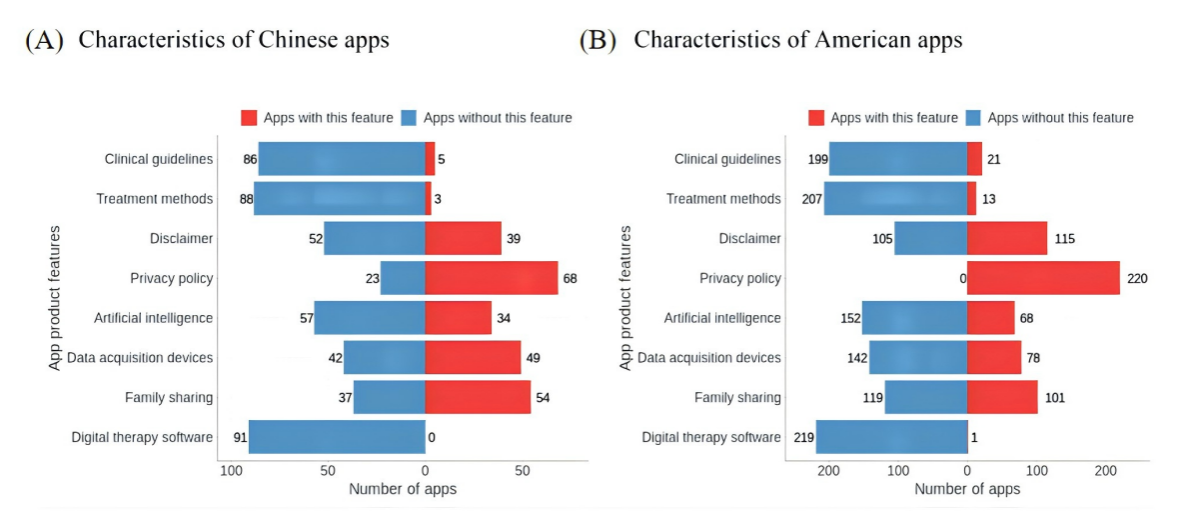
Product feature distribution of hypertension management apps in the US and China.

Standardization and technical perfection are the 2 main external product characteristics of HMAs. Detailed external feature information for the 261 HMAs and the results summary are presented in Table S6 in Multimedia Appendix 1. Regarding app development standardization, 237 apps (90.8%) did not adhere to clinical guidelines for hypertension, 246 apps (94.3%) did not use specific diagnostic and treatment methods for blood pressure management, 134 apps (51.3%) did not provide disclaimers, and 238 apps (91.2%) disclosed their data privacy policies to users. Regarding the apps’ technical completeness, 88 apps (33.7%) used artificial intelligence technology, with 86 (33.0%) using rule-based algorithms. Additionally, 105 apps (40.2%) required connections to specific data acquisition devices, with blood pressure monitors (n=79, 30.3%) and wearable devices (n=26, 10.0%) being the most commonly used. Moreover, 120 apps (46.0%) supported family blood pressure monitoring and data sharing, while only 1 app (0.4%) was classified as digital therapy software [23]. Regarding differences in product features between apps in the United States and China, most HMAs listed in Chinese app stores required specific external devices, and there was a greater emphasis on supporting family blood pressure monitoring and data sharing (Figure 5).

#### App Downloads and User Ratings

All HMAs were downloaded a total of 171,074,013 times from major app stores. The most downloaded app was the Blood Pressure App developed by QR Code Scanner, with over 50 million downloads. By contrast, the least downloaded app had only a few hundred downloads. On average, each app was downloaded 655,455 times. Most apps had fewer than 10,000 downloads (n=85, 32.6%) or between 10,000 and 100,000 downloads (n=74, 28.4%). For apps listed in Chinese app stores, there were 24,204,832 downloads, with an average of 265,987 downloads per app. For apps listed in US app stores, the total number of downloads was 148,869,181, averaging 676,678 downloads per app. There was no significant difference in the number of downloads between apps listed in the US and Chinese app stores (t_309_=–1.044, *P*=.30).

Further, all HMAs received a total of 1,721,889 ratings. Apps listed in Chinese app stores received 104,986 ratings, while those listed in American app stores received 1,616,903 ratings. Regarding user review ratings, 4- and 5-star ratings accounted for 75.22% (87,773/116,686) of all ratings, with Chinese apps receiving 83.72% (13,866/16,561) 4- and 5-star ratings and American apps receiving 73.81% (73,907/100,125).

### Functional Availability Evaluation of Hypertension Management Apps

This study systematically summarized clinical guidance documents on hypertension from the United States, China, and relevant international organizations, identifying a total of 26 relevant documents: 9 from the United States, 14 from China, 2 from the World Health Organization, and 1 from the International Society of Hypertension (see Table S7 in Multimedia Appendix 1). We summarized the specific functions of 261 HMAs and identified 9 functional feature dimensions and 50 specific functions (see the “Functional Availability of Hypertension Management Apps” section in Multimedia Appendix 1 for detailed results, Table S8 in Multimedia Appendix 1 for business needs, Table S9 in Multimedia Appendix 1 for functions, and Figure S2 in Multimedia Appendix 1 for functional characteristics). For overall app availability, there are 10 dimensions of business needs for hypertension management in China. The functions of the Chinese HMAs can meet 9 of these demand dimensions proposed in the Chinese Hypertension Clinical Guidance Document, with the exception of the measurement/treatment effect evaluation demand dimension, which remains unmet. The feature functions provided by HMAs meet 40 specific needs, with 15 specific needs remaining unmet. These unmet needs include adverse reaction monitoring, health behavior intervention, and follow-up treatment, such as recording adverse reactions, smoking cessation intervention, drug intervention, and follow-up for patients with hypertension. The business requirements for hypertension management in the United States encompass 12 dimensions. HMA functions in the United States can meet the 9 demand dimensions outlined in the clinical guidelines for hypertension in the United States. However, 3 demand dimensions—measurement/treatment efficacy evaluation, treatment plan reliability, and software performance—were not addressed. The feature functions effectively meet 39 specific needs, but 12 specific needs remain unmet. These specific unmet needs include personalized treatment for patients, classification of hypertension causes, and evaluation of treatment effects. Examples of these unmet needs are setting blood pressure control goals tailored to different patients, classifying hypertension phenotypes, and evaluating the effects of antihypertensive treatments.

**Figure 6:**
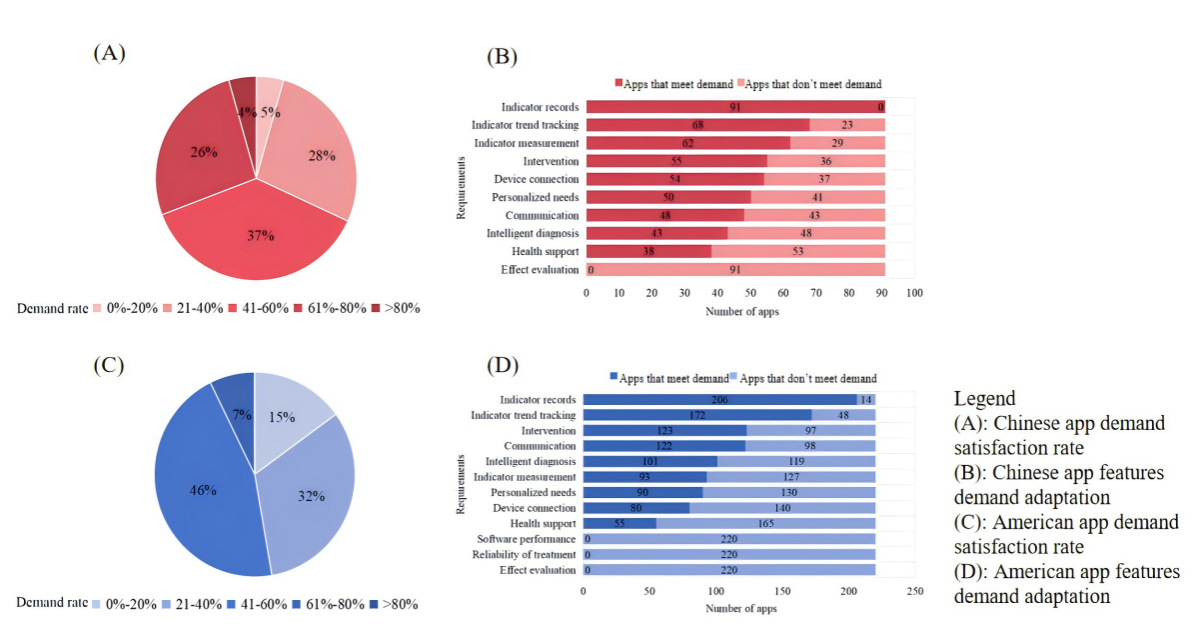
Distribution of demand satisfaction rate and features-demand adaptation of hypertension management apps in the US and China.

For single-app functionality, the average functional utilization rate of Chinese HMAs was 100% (91/91), but the average demand satisfaction rate was only 55.9%. Additionally, 37% (34/91) of Chinese apps had a demand satisfaction rate between 41% and 60%, while only 4 apps (4%, 4/91) had a demand satisfaction rate exceeding 80% (Figure 6A). Summarizing the satisfaction of each demand dimension in Chinese apps, the vast majority were able to meet the demand dimensions for health indicator recording (91/91, 100%), health indicator trend tracking (68/91, 75%), and health indicator measurement (62/91, 68%). However, the dimensions of measurement/treatment effect evaluation (0/91, 0%), health support (38/91, 42%), and intelligent diagnosis of hypertension (43/91, 47%) were not effectively met (Figure 6B). The average functional utilization rate of HMAs in the United States was 100% (220/220), but the average demand satisfaction rate was only 39.47% (86.83/220). Additionally, 45.4% (100/220) of American apps had a demand satisfaction rate of 41%-60%, and 15% (33/220) had a rate of 0%-20%. No American app had a demand satisfaction rate exceeding 80% (Figure 6C). Regarding the satisfaction status of each demand dimension in American apps, most American apps meet the demand dimensions of health indicator recording (206/220, 93.6%), health indicator trend tracking (172/220, 78.2%), and health intervention measures (123/220, 55.9%). However, the dimensions of measurement/treatment effect evaluation, treatment plan reliability, and software performance were not met (Figure 6D).

### User Satisfaction Evaluation Indicator System for Hypertension Management Apps

**Figure 7:**
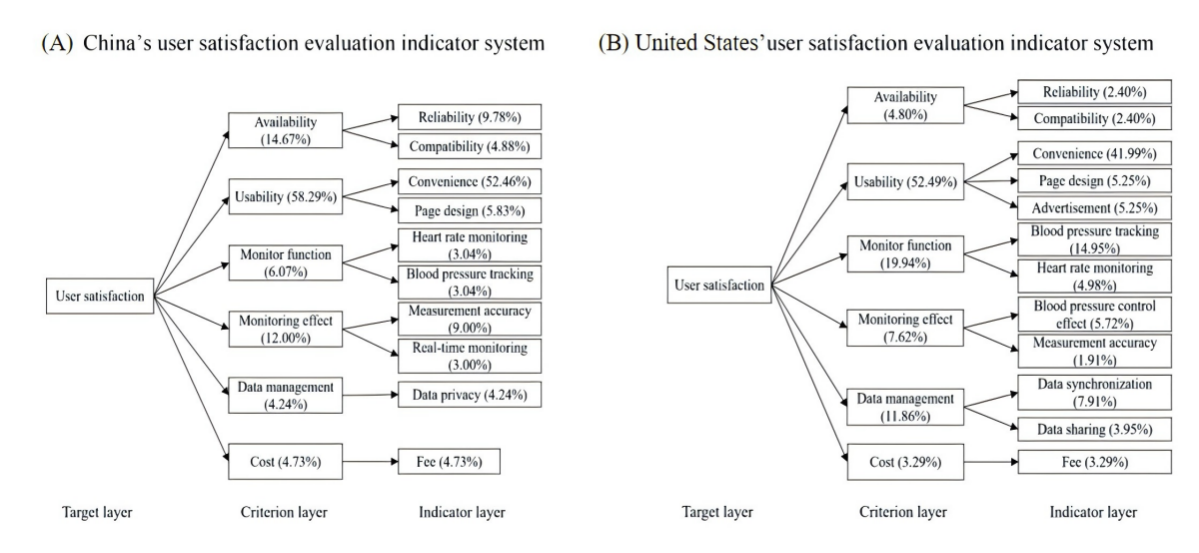
User satisfaction evaluation indicator systems in the United States and China.

In the previous study [23], 10 out of 12 significant factors were found to affect user satisfaction with HMAs in both China and the United States. Based on these factors, this study developed a framework for evaluating user satisfaction in both countries (see Figure S3 in Multimedia Appendix 1). Using the proportions of themes corresponding to various influencing factors in user reviews from the United States and China (see Table S10 in Multimedia Appendix 1), we applied AHP to calculate the weights of each indicator in the user satisfaction framework for both countries. The results are detailed in Tables S11-S16 in Multimedia Appendix 1. Using the indicator system framework and calculated weights, we constructed the USEISs for HMAs in the United States and China (Figure 7). In the US evaluation indicator system, the top 3 comprehensive weighted indicators were convenience (41.99%), blood pressure tracking (14.95%), and data synchronization (7.91%). In the Chinese USEIS, the top 3 indicators were convenience (52.46%), reliability (9.78%), and measurement accuracy (9.00%). The process of developing the framework, calculating indicator weights, and performing consistency checks using the AHP for HMA user USEISs in the United States and China is detailed in the “User Satisfaction Evaluation Indicator System for Hypertension Management App” section in Multimedia Appendix 1.

### User Satisfaction Evaluation Results of Hypertension Management Apps

This study used the proportion of positive reviews corresponding to each indicator as the initial satisfaction value and assessed overall user satisfaction and specific satisfaction with each criterion for both American and Chinese users, based on the importance weights in the 2 USEISs (see Figures S4-S7 in Multimedia Appendix 1). The evaluation results are presented in Tables 1 and 2. Among them, Chinese users had relatively low overall satisfaction with HMAs (evaluation value = 0.7891 out of 1). At the criteria layer, users expressed higher satisfaction with software usability (0.8721) and software monitoring functions (0.8243), while their satisfaction with cost (0.4303) and software monitoring effectiveness (0.6305) was relatively low. In the indicator layer, American users were relatively satisfied with convenience (0.8909) and heart rate monitoring (0.8358), while their satisfaction with cost (0.4303) and compatibility (0.4419) was relatively low. The overall satisfaction level of American users with HMAs was lower than that of Chinese users (0.6861 vs 0.7891). At the criteria layer, users had higher satisfaction with software monitoring effectiveness (0.8457) and cost (0.8093), but their satisfaction with data management (0.3163) and software availability (0.3647) was relatively low. In the indicator layer, users were relatively satisfied with the effectiveness of blood pressure management (0.8701), page design (0.8552), blood pressure tracking (0.8347), and cost (0.8093). However, their satisfaction with advertising distribution (0.2058), data synchronization (0.2145), and reliability (0.2977) was relatively low.

**Table 1:** Comprehensive satisfaction evaluation results of users of the US hypertension management apps.

Criterion layer (criteria satisfaction) and indicator layer^a,b^			Indicator satisfaction
**Availability (0.3647)**			
	Reliability	0.2977	
	Compatibility	0.4316	
**Usability (0.7353)**			
	Convenience	0.7865	
	Page design	0.8552	
	Advertising distribution	0.2058	
**Monitoring function (0.7726)**			
	Blood pressure tracking	0.8347	
	Heart rate monitoring	0.5863	
**Monitoring effect (0.8457)**			
	Effect of blood pressure management	0.8701	
	Measurement accuracy	0.7723	
**Data management (0.3163)**			
	Data synchronization	0.2145	
	Data sharing	0.5200	
**Cost (0.8093)**			
	Fee	0.8093	

^a^Target layer: user satisfaction.

^b^Overall satisfaction: 0.6861.

**Table 2:** Comprehensive satisfaction evaluation results of users of the Chinese hypertension management apps.

Criterion layer (criteria satisfaction) and indicator layer^a,b^			Indicator satisfaction		
**Availability (0.7044)**					
	Reliability	0.8356			
	Compatibility	0.4419			
**Usability (0.8721)**					
	Convenience	0.8909			
	Page design	0.7028			
**Monitoring function (0.8243)**					
	Heart rate monitoring	0.8358			
	Blood pressure tracking	0.8127			
**Monitoring effect (0.6305)**					
	Measurement accuracy	0.6188			
	Real-time monitoring	0.6656			
**Data management (0.7396)**					
	Data privacy	0.7396			
**Cost (0.4303)**					
	Fee	0.4303			

^a^Target layer: user satisfaction.

^b^Overall satisfaction: 0.7891.

## Discussion

### Product Development and User Usage Status of Hypertension Management Apps

In this study, although the number of HMAs increased in both the United States and China, there were significant differences in the number and frequency of app usage between the 2 countries. The overall number of HMAs launched has been increasing each year. However, the total number of HMAs in China (n=91) and the annual number of listings (n=6) were both lower than those in the United States (n=220 and n=15, respectively). This discrepancy may be related to differences in hypertension management practices between the 2 countries. In China, there is a greater emphasis on hospital visits and follow-ups, whereas the United States promotes self-health management by patients. As a result, American users are more likely to utilize HMAs. Additionally, most HMAs do not adhere to hypertension clinical guidelines, lack disclaimers, exhibit low levels of intelligence, and have inadequate standardized review processes. It is recommended to improve the standardization of app development by integrating standard clinical guidelines, enhancing the app’s intelligence with new wearable devices and artificial intelligence technologies, clarifying regulatory approval policies for digital therapy software for chronic diseases, and refining the approval process. Additionally, while the overall usage frequency of HMAs is relatively high, further efforts to address these areas could enhance their effectiveness and user satisfaction. Although the usage frequency of HMAs among Chinese users was significantly lower compared with American users, the average usage frequency for each type of HMA did not differ substantially between the 2 countries. The total downloads of all HMAs exceeded 170 million, with over 140 million downloads in the United States and around 20 million in China. There was no significant difference in the average downloads per app between the 2 countries. This indicates that both American and Chinese users generally have positive attitudes toward HMA use. The lower total download rate among Chinese users may be attributed to the smaller number of HMA users in China.

### Availability of Hypertension Management Apps

HMAs have diverse business demands but often exhibit poor functional availability. The summary of clinical guidelines for hypertension in the United States and China reveals that the business needs of HMAs in the United States emphasize the reliability of treatment plans and app performance, while in China, the focus is more on blood pressure monitoring and intelligent diagnosis. This may be related to the differing usage habits of users in the United States and China. US users prioritize ensuring the safety and effectiveness of apps, while Chinese users focus on comprehensive self-management of hypertension. Although HMAs currently offer some basic functions, their functional availability is still inadequate and does not fully address most needs for hypertension management. The summary of app functions reveals that most HMAs can assist users with recording blood pressure and heart rate, dynamically tracking blood pressure trends, and providing health interventions, thus addressing the need for blood pressure recording and health support. However, current apps still lack features such as intelligent hypertension diagnosis, personalized services, and health behavior interventions. HMAs must address business needs including blood pressure classification, blood pressure warnings, personalized treatment for different patient groups, family health management, and health behavior management.

### User Satisfaction Evaluation Indicator System for Hypertension Management Apps

The results of HMA USEIS for the United States and China indicate that while the evaluation criteria for app user satisfaction are consistent across both countries, there are significant differences in the weighting of these criteria. This discrepancy may be attributed to variations in app performance attributes and the differing usage habits of HMA users in China and the United States. Although the performance attributes of apps in both countries are consistent, the differing usage purposes and habits lead to variations in the weighting of evaluation criteria. Chinese users prioritize usability, functionality, and monitoring effectiveness, while American users focus more on usability, monitoring functions, and data management. Additionally, there are significant differences in the specific evaluation indicators and their importance weights. This may be related to differences in the demand for hypertension management between users in the United States and China. Clear operating procedures and user-friendly designs are fundamental needs for users in both countries and simple, intuitive apps are appealing. Chinese users primarily use apps for blood pressure measurement and recording, which leads to higher requirements for the app’s reliability and measurement accuracy. If an app frequently crashes or has significant measurement errors, it directly impacts its usability. American users primarily use apps for comprehensive blood pressure management, which includes data collection from wearable devices, real-time data synchronization, blood pressure recording, and visualization of blood pressure charts. Therefore, robust blood pressure tracking features and effective data synchronization are crucial for user satisfaction. Additionally, both American and Chinese users reported low overall satisfaction with HMAs (0.6861 vs 0.7891, respectively), which may be attributed to the inadequate functionality of HMAs in meeting user expectations. Most users expect HMAs to support comprehensive self-management of hypertension throughout their entire life cycle, including blood pressure measurement, recording, visual chart display, health behavior intervention, drug intervention, and intelligent diagnosis. However, most HMAs offer only limited functionality, failing to meet all these needs and resulting in low user satisfaction.

### Suggestions for Improving User Satisfaction of Hypertension Management Apps

#### Improve Product Functionality According to Clinical Guidelines

For Chinese HMAs, it is recommended to enhance functions such as health communities, intelligent diagnosis of hypertension, and doctor-patient communication. Building an online health community for patients with hypertension is crucial. This involves strengthening communication and exchange among users, providing additional emotional support, and enhancing their engagement with the app. Second, using artificial intelligence to develop a diagnostic algorithm for hypertension based on users’ personal health data is recommended. This approach can help users quickly assess their hypertension risk or determine its severity. Third, establishing a doctor-patient communication platform is recommended. This platform would allow users to have online consultations, enable doctors to manage patients remotely, strengthen doctor-patient interactions, and reduce unnecessary procedures.

For HMAs in the United States, it is recommended to enhance functions such as family health management, external device connectivity, and personalized patient management. First, developing a family blood pressure management feature can enable real-time monitoring of blood pressure indicators and provide early warnings for abnormalities, particularly for older family members. Second, enhancing the use of smart wearable devices and blood pressure monitors will improve the app’s data synchronization capabilities, enabling real-time monitoring of blood pressure indicators. Third, in line with American clinical guidelines for hypertension, it is important to develop personalized management functions. This includes tailored diagnosis and treatment for patients with various types of hypertension or hypertension with different complications.

#### Strengthen Monitoring and Improvement of User Satisfaction With a Hypertension Management App

Suggestions for strengthening the monitoring and improvement of user satisfaction are based on the HMA satisfaction evaluation indicator system. This involves real-time monitoring and evaluation of user satisfaction according to indicator weights and satisfaction ratings, followed by targeted improvements for factors associated with lower satisfaction. For example, American users have lower satisfaction with HMA advertising distribution, data synchronization, and reliability, with data synchronization being a factor with higher importance weights. This suggests that HMAs in the United States should prioritize improving data synchronization, optimizing advertising distribution, and enhancing app reliability. Chinese users reported poor satisfaction with cost, compatibility, and measurement accuracy, with measurement accuracy being a highly important factor. This indicates that Chinese HMAs need to focus on addressing inaccurate measurement results and, in addition, resolve issues related to high fees and poor app compatibility.

#### Increase Policy and Financial Support for Hypertension Management Apps

It is imperative to provide companies with entrepreneurial subsidies, establish expedited review channels for digital health software aimed at chronic disease management, and promote collaboration between companies and hospitals. Additionally, increasing funding for chronic disease digital health software and offering financial support for HMA development are recommended.

### Limitations

This study had some limitations. First, due to differences in health care policies, diagnoses, and treatment models between the United States and China, the quantity and frequency of HMA use in China are lower than in the United States. To ensure data representativeness, the study did not use specialized sampling but instead included all available apps and user reviews. The volume of data collected was sufficient to accurately reflect the situation of HMAs in the United States and China. Second, while this study identified differences in the USEISs between the 2 countries and provided a preliminary analysis of these differences, a more in-depth analysis is needed. Finally, future research should focus on the following aspects: First, obtaining nonsensitive user data from app developers, such as age, sex, patient status, and whether the app is used personally, could further elucidate how different user characteristics impact satisfaction. Second, exploring how different data acquisition methods (eg, automatic collection from devices such as blood pressure monitors versus manual input by users) affect satisfaction is valuable. Third, further research should investigate the relationship between user satisfaction and continuance intention, focusing on factors influencing continuance intention and their specific pathways. Fourth, as users are increasingly concerned with therapeutic outcomes, future studies should integrate clinical efficacy data with user satisfaction to better understand the relationship between user satisfaction and clinical effectiveness.

### Conclusions

Research indicates that both the functional availability and user satisfaction with HMAs in the United States and China are lacking. The current app functions fail to meet all core business needs, and there are notable differences in the USEISs between the 2 countries. The key indicators in the evaluation system for US HMAs are convenience, blood pressure tracking, and data synchronization. By contrast, the most important indicators for Chinese HMAs are convenience, reliability, and measurement accuracy. These findings offer valuable insights for app developers to enhance product performance and boost user satisfaction. Additionally, they assist users in selecting apps that best meet their needs.

## Appendix

Multimedia Appendix 1 Supplementary materials.

## Abbreviations

AHP: analytic hierarchy process
HMA: hypertension management app
TTF: task-technology fit
USEIS: user satisfaction evaluation indicator system

## Data Availability

This study obtained all HMA and user reviews from 8 Chinese app stores (China Apple App Store, Huawei App Store, Xiaomi App Store, Oppo App Store, Vivo App Store, Baidu App Store, 360 App Store, and Application Treasure App Store) and 2 English app stores (US Apple App Store and US Google Play Store) through web scraping technology and the Qimai app data official website. The crawler code can be found in [62]. The code for latent Dirichlet allocation topic modeling and statistical analysis in the author’s previous study [23] can be found in [63]. The code for calculating the weight of indicators using the AHP can be found in [64].
