# Supplementary material for: Establishing an Evaluation Indicator System for User Satisfaction With Hypertension Management Apps: Combining User-Generated Content and Analytic Hierarchy Process
Source: J Med Internet Res. 2024 Sep 3;26:e60773. doi: 10.2196/60773 (PMC11408894; doi:10.2196/60773)
Supplement: Multimedia Appendix 1 [file jmir_v26i1e60773_app1.docx]

# Multimedia Appendix 1

**For Manuscript:**

**“Establishing an Evaluation Indicator System for User Satisfaction With Hypertension Management Apps: Combining User-Generated Content and Analytic Hierarchy Process”**

# 1 Obtaining, screening, and preprocessing of hypertension management apps and their user reviews

## 1.1 Access and screening of hypertension management apps in the United States and China

In April 2023, we used 10 Chinese keywords such as “blood pressure”, “hypertension”, and “blood pressure management” to search for the Chinese hypertension management app (HMA) in 8 Chinese app stores, including the China Apple app store, Huawei app store, Xiaomi app store, OPPO app store, VIVO app store, Baidu app store, 360 app store, and Application treatment app store. We also used the two English keywords “hypertension” and “high blood pressure” to search for the US HMA in the US Apple app store and US Google play store. Finally, a total of 5016 HMAs were retrieved for screening, with 3591 apps remaining after deduplication (detailed search terms and results can be found in Table 1). In addition, to further identify the HMA, we arranged for two researchers (YFH and JL) to screen separately based on mature inclusion and exclusion criteria (the two researchers received unified training and had good consistency before screening, kappa=0.84), and their differences were arbitrated and resolved by another cardiovascular clinical expert (HC). Finally, a total of 261 HMAs with user reviews were included, of which 41 apps were only listed on Chinese app stores, 170 apps were only listed on American app stores, and 50 apps were simultaneously listed on Chinese and American app stores (detailed inclusion and exclusion criteria and screening process diagrams are shown in Table 2 and Figure 1, respectively).

## 1.2 Preprocessing of user reviews

We used the Qimai mobile application data analysis platform and self-developed python scripts to obtain user reviews and corresponding user ratings for 261 HMAs included in the study. As of April 23, 2023, a total of 295,927 reviews and corresponding rating data from Chinese and American users have been obtained, including 250,193 reviews from American users and 45,734 reviews from Chinese users. To further ensure the authenticity and reliability of user reviews, we have carried out the following preprocessing. Firstly, we used the R’s tweetbotnot package to identify robot accounts based on account characteristics such as registration time, number of reviews, and number of deleted reviews, and deleted all reviews posted by the robot account. 70,371 reviews posted by robot accounts were removed (including 53,431 reviews from US users and 16,940 reviews from Chinese users). Secondly, automatically remove 43,418 duplicate reviews (including 35,408 reviews from US users and 8010 reviews from Chinese users). Then, we manually identified invalid user reviews, including garbled characters, emoticons, non Chinese or English reviews, and meaningless reviews such as advertisements. 47,108 invalid reviews (including 45,421 US user reviews and 1687 Chinese user reviews) were manually identified and deleted. Furthermore, the SKEP sentiment pre training algorithm was used to calculate the sentiment polarity of each user review, and 10,605 reviews with conflicting sentiment polarity and ratings were removed (including 9243 US user reviews and 1362 Chinese user reviews). Finally, remove 7739 blank reviews after text segmentation and removal of stop words (including 6565 reviews from US users and 1174 reviews from Chinese users). Finally, this study included 116,686 Chinese and American user reviews to construct a user satisfaction evaluation indicator system, including 100,125 American user reviews (accounting for 0.067% of the total app downloads) and 16,561 Chinese user reviews (accounting for 0.068% of the total app downloads) (the number of HMAs and user reviews listed on each app store is shown in Table 3).

# 2 Summary of app business requirements

## 2.1 Clinical guidelines for hypertension and expert consensus sorting

Considering the significant differences in hypertension management models and business needs among populations in different periods and countries, this study selected the United States and China as the research subjects, and summarized the latest clinical guidance documents on hypertension management, diagnosis and treatment in both countries. The hypertension guidance guidelines from the world health organization and the international society of hypertension were collected as supplements. Firstly, search and obtain clinical guidelines, clinical practices, expert consensus, and dietary guidelines for hypertension management and diagnosis in both the United States and China from their official websites. Secondly, use keywords such as “hypertension”, “guidelines”, “clinical practice”, and “expert consensus” to search for relevant English and Chinese literature in the Chinese National Knowledge Infrastructure, Web of Science, and PubMed literature databases, and identify guidance literature published by hypertension management organizations in the United States and China from these literature. Next, consult clinical experts (HC) in the field of cardiovascular to supplement the missing clinical guidance documents for hypertension in the United States and China. Then, find the international hypertension management guidelines from the official websites of the World Health Organization and the International Society for Hypertension as a supplement to the clinical guidelines for hypertension in the United States and China. Finally, the documents published by official institutions in the United States and China on their official websites, relevant guidance literature published in English and Chinese journals, expert supplementary documents, and international hypertension management guidance documents are summarized into clinical guidance documents for hypertension in the United States and China, respectively. The characteristic information of these clinical guidance documents, such as the country/institution of publication, publication date, file name, publishing unit, file type, guidance content, and original text link, are extracted and summarized.

## 2.2 Summary of business needs for hypertension management

This study arranged for a clinical expert (HC) in the field of cardiovascular to systematically review the clinical guidance documents for hypertension in the United States and China, and summarize the business needs of users in both countries for hypertension management. Firstly, carefully read all clinical guidance documents for hypertension in the United States and China, and extract the requirements and suggestions for hypertension management contained in each guidance document, such as blood pressure monitoring, dietary intervention, exercise intervention, and hypertension follow-up. Then, summarize all the business requirements for hypertension management in the clinical guidance documents of the United States and China respectively, remove duplicate requirements in each document, and classify and organize these requirements to form a collection of business requirements for hypertension management for both American and Chinese users.

# 3 Functional availability of hypertension management apps

## 3.1 The business needs for hypertension management

This study systematically summarized clinical guidance documents on hypertension from the United States, China, and relevant international organizations. The summary results are shown in Table 7. These guidance documents include various types of documents such as clinical guidelines, expert consensus, survey reports, scientific statements, clinical practices, and dietary guidelines, comprehensively covering different aspects of hypertension management such as self-management, dietary intervention, medication management, diagnosis and treatment, and blood pressure monitoring. Among them, the United States has 9 clinical guidance documents related to hypertension management, China has 14 related documents, the World Health Organization has 2 related documents, and the International Society of Hypertension has 1 related document.

Extract and summarize the business requirements for hypertension management in the United States and China from the above clinical guidance documents. The summary results are shown in Table 8. Among them, there are 10 dimensions of business needs for hypertension management in China, including indicator measurement, indicator recording, indicator trend tracking, device connection, health intervention measures, personalized needs, intelligent diagnosis of hypertension, measurement/treatment effect evaluation, doctor-patient communication, and health support. A total of 55 specific needs are included in these 10 dimensions. The business requirements for hypertension management in the United States have 12 dimensions. In addition to the 10 business requirements in China, there is also an increased focus on the reliability and software performance of treatment plans, such as the maturity, software reliability, and usability of app treatment plans. A total of 51 specific requirements are included in these 12 dimensions.

## 3.2 Summary of the main functions of the app

261 HMAs contain a total of 9 functional feature dimensions and 50 specific functions, as detailed in Table 9. For the functional feature dimension of HMA, 247 (94.64%) apps have relevant functions for recording health indicators, such as blood pressure recording (n=243 [93.10%]) and heart rate recording (n=181 [69.35%]). 198 apps (75.86%) can track changes in different health indicators, among which 187 apps (71.65%) and 111 apps (42.53%) can dynamically track changes in blood pressure and heart rate through line charts and other forms, respectively. In addition, most apps also have functional dimensions such as health intervention (n=150 [57.47%]), indicator measurement (n=144 [55.17%]), doctor-patient communication (n=140 [53.64%]), intelligent diagnosis (n=126 [48.28%]), and device connectivity (n=109 [41.76%]), while there are relatively few apps that can achieve personalized services (n=104 [39.85%]) and health support (n=75 [28.74%]). For the specific functions of HMA, 70.50% of apps only contain 10 or fewer specific functions. Blood pressure recording (n=243 [93.10%]), blood pressure trend tracking (n=187 [71.65%]), heart rate recording (n=181 [69.35%]), blood pressure data sharing (n=120 [45.98%]), and heart rate trend tracking (n=111 [42.53%]) are the basic functions that most apps have, while there are relatively few apps with core functions of hypertension management, such as blood pressure measurement (n=82 [31.42%]), drug management (n=49 [18.77%]), dietary intervention (n=39 [14.94%]), exercise intervention (n=35 [13.41%]), blood pressure classification (n=76 [29.12%]), and blood pressure warning (n=28 [10.73%]).

The functional feature dimensions contained in HMA in the United States and China are the same, but there are significant differences in their specific distribution, as shown in Figure 2. Both US and Chinese apps have 9 identical functional feature dimensions, and indicator recording, indicator trend tracking, indicator measurement, and health intervention are the basic functional dimensions that most apps have. However, in addition to basic functional dimensions, Chinese apps pay more attention to device connectivity (n=54 [59.34%]) and personalized services (n=50 [54.95%]), such as automatic data uploading for wearable devices and incentives for healthy behavior. American apps focus more on doctor-patient communication (n=122 [55.45%]) and intelligent diagnosis of hypertension (n=101 [45.91%]), such as online consultations and blood pressure classification.

# 4 User satisfaction evaluation indicator system for hypertension management app

## 4.1 Framework of user satisfaction evaluation indicator system

The author of this article found 10 significant factors that affect user satisfaction with Chinese HMA in the preliminary study, including reliability, compatibility, convenience, page design, heart rate monitoring, blood pressure tracking, measurement accuracy, real-time monitoring, data privacy, and cost. 12 significant factors affecting user satisfaction with American HMA were identified, including reliability, compatibility, convenience, page design, advertising distribution, blood pressure tracking, heart rate monitoring, blood pressure management effectiveness, measurement accuracy, data synchronization, data sharing, and cost. Meanwhile, the proportion of these influencing factors corresponding to themes in user reviews in the United States and China was summarized, as shown in Table 10. Based on these significant influencing factors, this study developed a framework for evaluating user satisfaction in the United States and China, as shown in Figure 3.

## 4.2 Importance weight of user satisfaction evaluation system indicators

The frequency of each indicator and criterion in the user satisfaction evaluation indicator system of the United States and China is shown in Table 11. Among them, the most frequent indicator in the evaluation indicator systems of both countries is convenience (34.40% in the United States and 41.44% in China), and the criterion is usability (38.39% in the United States and 44.28% in China). The importance scale results of the 1-9 levels of user satisfaction evaluation indicator systems in the United States and China are shown in Table 12. Among them, the unit importance scale of the evaluation indicator system in the United States is 4.20%, indicating that for every 4.20% increase in the frequency ratio difference between one indicator and another, its importance scale increases by 1. The unit importance scale of China's evaluation indicator system is 4.79%, indicating that for every 4.79% increase in the frequency ratio difference between one indicator and another, its importance scale increases by 1.

For the calculation of indicator weights in the US user satisfaction evaluation indicator system, first, perform hierarchical single ranking and consistency testing. Construct a judgment matrix for the criterion layer based on the importance scale of the US user satisfaction evaluation indicator system, calculate the importance weights of each criterion through hierarchical single sorting, and verify the consistency of the results. The results are shown in Figure 4. The standardized maximum eigenvectors of the criterion layer judgment matrix are [0.0480, 0.5249, 0.1994, 0.0762, 0.1186, 0.0329], and the consistency coefficient CR value is 0.0297, indicating that consistency testing has been conducted. Afterwards, a judgment matrix was constructed for each indicator in the 6 criteria, and the importance weights of each indicator were calculated through hierarchical single sorting. The consistency of the results was verified, as shown in Figure 5. Among them, the standardized maximum eigenvectors of the availability judgment matrix are [0.5, 0.5], the standardized maximum eigenvectors of the usability judgment matrix are [0.8, 0.1, 0.1], the standardized maximum eigenvectors of the monitoring function judgment matrix are [0.75, 0.25], the standardized maximum eigenvectors of the monitoring effect judgment matrix are [0.75, 0.25], the standardized maximum eigenvectors of the data management judgment matrix are [0.6667, 0.3333], and the standardized maximum eigenvectors of the cost judgment matrix are [1]. All matrices are consistency matrices. Then, perform a hierarchical total ranking and consistency check. Based on the importance weights of the criterion layer and indicator layer, the comprehensive weights of each indicator on user satisfaction are calculated using a linear weighted sum method, and combined consistency and overall consistency tests are performed. The results are shown in Tables 13,14. Among them, convenience (41.99%), blood pressure tracking (14.95%), and data synchronization (7.91%) are the top three comprehensive weight indicators in the US user satisfaction evaluation indicator system. In addition, the combination consistency coefficient and overall consistency coefficient of each level are both less than 0.1, indicating that the consistency test has been passed.

For the calculation of indicator weights in the Chinese user satisfaction evaluation indicator system, first, perform hierarchical single ranking and consistency testing. Construct a judgment matrix for the criterion layer based on the importance scale of the Chinese user satisfaction evaluation indicator system, calculate the importance weights of each criterion through hierarchical single sorting, and verify the consistency of the results. The results are shown in Figure 6. The standardized maximum eigenvectors of the criterion layer judgment matrix are [0.1467, 0.5829, 0.0607, 0.1200, 0.0424, 0.0473], and the consistency coefficient CR value is 0.0268, indicating that consistency testing has been conducted. Afterwards, a judgment matrix was constructed for each indicator in the 6 criteria, and the importance weights of each indicator were calculated through hierarchical single sorting. The consistency of the results was verified, as shown in Figure 7. Among them, the standardized maximum eigenvectors of the availability judgment matrix are [0.6667, 0.3333], the standardized maximum eigenvectors of the usability judgment matrix are [0.9, 0.1], the standardized maximum eigenvectors of the monitoring function judgment matrix are [0.5, 0.5], the standardized maximum eigenvectors of the monitoring effect judgment matrix are [0.75, 0.25], and the standardized maximum eigenvectors of the data management judgment matrix and the cost judgment matrix are both [1]. All matrices are consistency matrices. Then, perform a hierarchical total ranking and consistency check. Based on the importance weights of the criterion layer and indicator layer, the comprehensive weights of each indicator on user satisfaction were calculated using a linear weighted sum method, and combined consistency and overall consistency tests were performed. The results are shown in Tables 15,16. Among them, convenience (52.46%), reliability (9.78%), and measurement accuracy (9.00%) are the top three comprehensive weighted indicators in the Chinese user satisfaction evaluation indicator system. In addition, the combination consistency coefficient and overall consistency coefficient of each level are both less than 0.1, indicating that the consistency test has been passed.

# 5 Summary of Tables

Table 1 Search terms and results for Chinese and American app stores

| Country | App store | Search term | Number of apps |
| --- | --- | --- | --- |
| China | China Apple app store | “血压”, “血压管理”, “血压控制”, “血压监测”, “血压测量”, “血压记录”, “高血压”, “高血压预防”, “高血压治疗”, “高血压随访”, “hypertension”, and “high blood pressure” | 566 |
|  | Huawei app store | “血压”, “血压管理”, “血压控制”, “血压监测”, “血压测量”, “血压记录”, “高血压”, “高血压预防”, “高血压治疗”, “高血压随访”, “hypertension”, and “high blood pressure” | 730 |
|  | Xiaomi app store | “血压”, “血压管理”, “血压控制”, “血压监测”, “血压测量”, “血压记录”, “高血压”, “高血压预防”, “高血压治疗”, “高血压随访”, “hypertension”, and “high blood pressure” | 475 |
|  | OPPO app store | “血压”, “血压管理”, “血压控制”, “血压监测”, “血压测量”, “血压记录”, “高血压”, “高血压预防”, “高血压治疗”, “高血压随访”, “hypertension”, and “high blood pressure” | 463 |
|  | VIVO app store | “血压”, “血压管理”, “血压控制”, “血压监测”, “血压测量”, “血压记录”, “高血压”, “高血压预防”, “高血压治疗”, “高血压随访”, “hypertension”, and “high blood pressure” | 538 |
|  | Baidu app store | “血压”, “血压管理”, “血压控制”, “血压监测”, “血压测量”, “血压记录”, “高血压”, “高血压预防”, “高血压治疗”, “高血压随访”, “hypertension”, and “high blood pressure” | 60 |
|  | 360 app store | “血压”, “血压管理”, “血压控制”, “血压监测”, “血压测量”, “血压记录”, “高血压”, “高血压预防”, “高血压治疗”, “高血压随访”, “hypertension”, and “high blood pressure” | 340 |
|  | Application treasure app store | “血压”, “血压管理”, “血压控制”, “血压监测”, “血压测量”, “血压记录”, “高血压”, “高血压预防”, “高血压治疗”, “高血压随访”, “hypertension”, and “high blood pressure” | 416 |
| The United States | US Apple app store | “hypertension”, and “high blood pressure” | 533 |
|  | US Google play store | “hypertension”, and “high blood pressure” | 895 |

Table 2 Detailed inclusion and exclusion criteria for hypertension management app

| Inclusion/exclusion criteria | Standard content |
| --- | --- |
| Inclusion criterion 1 (IC1) | The search terms were “血压”, “血压管理”, “血压控制”, “血压监测”, “血压测量”, “血压记录”, “高血压”, “高血压预防”, “高血压治疗”, “高血压随访”, “hypertension”, and “high blood pressure” for China app store and “hypertension”, and “high blood pressure” for US app store. |
| Inclusion criterion 2 (IC2) | Belonging to either health or medical apps. |
| Inclusion criterion 3 (IC3) | Must included apps for the blood pressure management, control, monitoring, measurement, recording, prevention, treatment or follow-up. |
| Inclusion criterion 4 (IC4) | The target users were those who need to control their blood pressure. |
| Exclusion criterion 1 (EC1) | Apps were only data receiving and transferring ends of external facilities or sensors and did not function for the control, monitoring, measurement, recording, prevention, treatment or follow-up of blood pressure. |
| Exclusion criterion 2 (EC2) | Apps have been removed from the app store. |
| Exclusion criterion 3 (EC3) | Blood pressure management is not the main function. |
| Exclusion criterion 4 (EC4) | The language used by the apps is not Chinese or English. |
| Exclusion criterion 5 (EC5) | Apps for no user review. |

Table 3 The number of hypertension management apps and user reviews on various app stores

| Country | App store | Number of apps included | Number of user reviews |
| --- | --- | --- | --- |
| China | China Apple app store | 64 | 2480 |
|  | Huawei app store | 30 | 2916 |
|  | Xiaomi app store | 20 | 4330 |
|  | OPPO app store | 16 | 1185 |
|  | VIVO app store | 20 | 2115 |
|  | Baidu app store | 9 | 606 |
|  | 360 app store | 8 | 2163 |
|  | Application treasure app store | 12 | 766 |
| The United States | US Apple app store | 119 | 25,616 |
|  | US Google play store | 129 | 74,509 |

Table 4 Feature information extraction framework for hypertension management app

| Product feature dimension | Product feature extraction fields | Descriptions/feature discrimination methods |
| --- | --- | --- |
| Basic information of the app | Application ID/Bundle ID | The software number of the app in the iOS or Android app store (for example, com. 1knet. RuiGuangKangTai) |
|  | App name | The software name of the app (e.g. daily blood pressure) |
|  | Development company | The name of the development company for the app (e.g. Shenzhen Yike Network Technology Co., Ltd.) |
| Listing situation | App store | 所有上架该APP的应用商店名称（例如，华为、小米和OPPO） |
|  | Listing time | The earliest listing time of this app in all app stores (e.g. February 14, 2015) |
|  | Latest update time | The latest update time for this app in all app stores (e.g. October 18, 2021) |
|  | Version iteration count | The app has the highest number of version updates among all app stores (e.g. 10) |
| User usage | Total downloads | The total number of downloads of this app across all app stores (e.g. 17500) |
|  | Total number of ratings | The total number of ratings for this app across all app stores (e.g. 81) |
| Development standardization | Does the development follow the clinical guidelines for hypertension | Based on the basic information description of the app in the app store, determine whether it follows the clinical guidelines for hypertension (including potential values of yes or no) |
|  | Does it rely on specific diagnostic and treatment methods | Determine whether the app store relies on specific diagnostic and treatment methods based on its app feature description (including potential values of yes or no) |
|  | Specific diagnostic and treatment methods used | If the app relies on specific diagnostic and treatment methods, list the specific diagnostic and treatment methods used (such as cognitive-behavioral therapy) |
|  | Is there a disclaimer | Determine whether there is a disclaimer based on the basic information description of the app in the app store (including potential values of yes or no) |
|  | Is there a privacy statement | Determine whether there is a user data privacy statement based on the basic information description of the app in the app store (including potential values of yes or no) |
| Technical completeness | Has artificial intelligence technology been used | Determine whether the app store has used artificial intelligence technology based on its app feature description (including potential values of yes or no) |
|  | Specific artificial intelligence technologies used | If the app uses artificial intelligence technology, list the specific artificial intelligence technology used (such as machine learning algorithms) |
|  | Are specific external devices for blood pressure monitoring connected | Determine whether a specific blood pressure monitoring external device needs to be connected based on the app feature description of the app store (including potential values of yes or no) |
|  | Specific external devices connected for blood pressure monitoring | If the app needs to connect to a specific external device for blood pressure monitoring, list the specific name of the external device for blood pressure monitoring (such as a blood pressure monitor) |
|  | Does it support family sharing | Determine whether the app store supports family data sharing based on its app feature description (including potential values of yes or no) |
|  | Does it belong to digital therapy | Determine whether the app belongs to digital therapy software based on its feature description in the app store (including potential values of yes or no) |

Table 5 Summary of hypertension management app functions

| Functional feature dimension | Specific functions | Functional interpretation |
| --- | --- | --- |
| Measurement of health indicators | Blood pressure measurement | Assist patients in measuring blood pressure |
|  | Heart rate measurement | Assist patients in measuring heart rate |
|  | Blood oxygen measurement | Assist patients in measuring blood oxygen levels |
|  | Sleep quality measurement | Assist patients in measuring sleep quality index |
|  | BMI calculation | Assist patients in calculating body mass index (BMI) |
|  | Temperature measurement | Assist patients in measuring body temperature |
|  | Psychological stress measurement | Measuring the psychological stress of patients through a scale |
| Record of health indicators | Blood pressure recording | Automatically obtain or manually input blood pressure values from external devices to view historical blood pressure values |
|  | Heart rate recording | Automatically obtain or manually input heart rate values from external devices to view historical heart rate values |
|  | Blood oxygen record | Automatically obtain or manually input blood oxygen values from external devices to view historical blood oxygen values |
|  | Sleep quality record | Automatically obtain or manually input sleep quality index from external devices to view historical sleep quality index |
|  | Blood glucose recording | Automatically obtain or manually input blood glucose values from external devices to view historical blood glucose values |
|  | Blood lipid record | Automatically obtain or manually input blood lipid values from external devices to view historical blood lipid values |
|  | Weight record | Manually recording patient's weight information |
|  | Thermometer recording | Manually recording patient's body temperature information |
|  | Electronic medical record management | Online input and management of personal medical records such as current and past medical history of hypertension |
| Trend tracking of health indicators | Visual display of blood pressure change trend | Visualize blood pressure trends in the form of line graphs and other forms |
|  | Visual display of heart rate change trend | Visualize heart rate trends in the form of line graphs and other forms |
|  | Visual display of blood glucose change trend | Visualize blood glucose trends in the form of line graphs and other forms |
|  | Visual display of blood oxygen change trend | Visualize the trend of blood oxygen in the form of line graphs and other forms |
|  | Electrocardiogram display | Draw an electrocardiogram based on measurement results |
| Health intervention measures | Blood pressure measurement reminder | Remind patients to measure blood pressure at specific times |
|  | Hypertension knowledge base | A knowledge guide for hypertension, helping patients understand and learn about hypertension management related knowledge |
|  | Health message push | Daily push of information on hypertension management and health management |
|  | Medication management | Medication advice, medication records, medication reminders, hypertension medication inquiries |
|  | Ischemic preconditioning training record/management | Improving patient blood pressure levels by planning and recording ischemic preconditioning training |
|  | Dietary records/management | Support recording and analyzing diet, and analyze nutrient compliance according to DASH guidelines |
|  | Sports recording/management | Record the number of steps taken, distance traveled, calories burned, recommend training courses, and set a training plan |
|  | Respiratory training | Improving heart health by completing respiratory training |
| Intelligent diagnosis of hypertension | Interpretation of measurement results | Interpret the measured blood pressure, heart rate, pulse and other numerical values or visual chart results, explain the actual meaning of the measured values or charts, and provide suggestions |
|  | Blood pressure classification | Classify blood pressure based on blood pressure values |
|  | Blood pressure warning | Intelligent assessment of blood pressure levels, determining whether the current blood pressure value is within the normal range, and providing early warning for abnormal values |
|  | Heart rate warning | Intelligent evaluation of heart rate level, determining whether the current heart rate value is within the normal range, and providing early warning for abnormal values |
|  | Psychological stress warning | Intelligent assessment of psychological stress levels, determining whether the current psychological stress level is within the normal range, and providing early warning for abnormal values |
|  | Online intelligent diagnosis | Intelligent diagnosis and evaluation of hypertension and cardiovascular risk levels |
| Doctor exchanges | Doctor-patient communication | Assist patients in communicating their condition online with their attending physician |
|  | Expert consultation | Online consultation with doctors or video interaction with doctors |
|  | Share blood pressure results report | Share blood pressure health results reports with doctors or other patients, or support exporting blood pressure health results reports |
| Device connection and data synchronization | Connecting a blood pressure monitor | Measure blood pressure by connecting a blood pressure monitor |
|  | Connect the smart bracelet | Real time monitoring and transmission of personal health data through connecting smart wristbands |
|  | Automatic synchronization of measurement data | Automatically synchronize the measured data to the software |
|  | Data backup | Backup and store data |
|  | Blood pressure meter calibration | Assist in accurate blood pressure measurement results |
| Health support | Online community | An online community among hypertensive patients, where patients can communicate about their condition |
|  | Family blood pressure health joint management | Family members can be added to contacts to jointly manage their blood pressure |
| Personalized/gamified features | Provide optional languages | Add language options such as Chinese, traditional Chinese characters, English, Spanish, Japanese, etc |
|  | Online shopping mall | Patients can purchase hypertension management equipment and medication online |
|  | Task check-in | Using gamified methods to clock in and promote patients to complete daily tasks such as blood pressure measurement, medication, diet, and exercise |
|  | Points mall | Provide check-in points function, which can be used to exchange items |
|  | Memorandum | Information such as medication experience, physical sensation, attending physician, and physical examination report that can be recorded by patients during the blood pressure management process |

Table 6 Product feature distribution of hypertension management app

| Characteristic dimension | Characteristic indicators | Eigenvalue | Number of app | Proportion |
| --- | --- | --- | --- | --- |
| Development standardization | Does it follow the clinical guidelines for hypertension | yes | 24 | 9.19% |
|  |  | no | 237 | 90.81% |
|  | Does it rely on specific diagnostic and treatment methods | yes | 15 | 5.75% |
|  |  | no | 246 | 94.25% |
|  | Is there a disclaimer | yes | 127 | 48.66% |
|  |  | no | 134 | 51.34% |
|  | Is there a data application privacy policy in place | yes | 238 | 91.19% |
|  |  | no | 23 | 8.81% |
| Technical completeness | Has artificial intelligence technology been used | yes | 88 | 33.72% |
|  |  | no | 173 | 66.28% |
|  | Whether to connect to specific data acquisition devices | yes | 105 | 40.23% |
|  |  | no | 156 | 59.77% |
|  | Does it support family sharing | yes | 120 | 45.98% |
|  |  | no | 141 | 54.02% |
|  | Is it a digital therapy software | yes | 1 | 0.38% |
|  |  | no | 260 | 99.62% |

Table 7 Summary of clinical guidance documents for hypertension

| Country | Release date | File name | Publishing unit | File type | Guidance content |
| --- | --- | --- | --- | --- | --- |
| China | 2010.03 | “Chinese Guidelines for the Prevention and Treatment of Hypertension, Third Edition (Revised in 2010)” | China Hypertension Alliance, etc | Clinical guidelines | Diagnosis and treatment of hypertension |
|  | 2019.02 | “Chinese Guidelines for the Prevention and Treatment of Hypertension (Revised 2018 Edition)” | China Hypertension Alliance, etc | Clinical guidelines | Diagnosis and treatment of hypertension |
|  | 2020.04 | “Consensus of Chinese Young and Middle aged Hypertension Management Experts” | Chinese Society of Cardiology | Expert consensus | Management |
|  | 2021.03 | “2020 edition of the National Guidelines for the Prevention and Treatment of Primary Hypertension” | National Cardiovascular Disease Center, etc | Clinical guidelines | Management |
|  | 2021.04 | “2020 China Ambulatory Blood Pressure Monitoring Guidelines” | China Hypertension Alliance | Clinical guidelines | Blood pressure monitoring |
|  | 2021.04 | “Multidisciplinary Expert Consensus on Heart Rate Management in Chinese Hypertensive Patients (2021 Edition)” | Multidisciplinary Consensus Group on Hypertensive Heart Rate Management | Expert consensus | Management |
|  | 2021.08 | “Expert Consensus on the Diagnosis and Treatment of Mental Stress Related Hypertension in Adults” | Chinese Medical Association | Expert consensus | Diagnosis and treatment of hypertension |
|  | 2022.05 | “2022 Taiwan Cardiology Association and Taiwan Hypertension Society Hypertension Management Guidelines” | The Taiwan Society of Cardiology, et al | Clinical guidelines | Diagnosis and treatment of hypertension |
|  | 2022.08 | “Chinese Expert Consensus on the Application of Intelligent Wearable Devices in Blood Pressure Management for Middle and Young People” | China Hypertension Alliance, etc | Expert consensus | Management |
|  | 2022.11 | “Chinese Clinical Practice Guidelines for Hypertension” | National Cardiovascular Disease Center, etc | Clinical guidelines | Diagnosis and treatment of hypertension |
|  | 2023.01 | “Adult Hypertension Dietary Guidelines (2023 Edition)” | Office of the National Health Commission | Dietary guidelines | Hypertensive diet |
|  | 2023.03 | “Key Points of the Chinese Hypertension Prevention and Treatment Guidelines (2023 Edition)” | China Hypertension Annual Conference | Clinical guidelines | Diagnosis and treatment of hypertension |
|  | 2023.05 | “Clinical Practice of Updating the 2023 Chinese Hypertension Prevention and Treatment Guidelines” | Yichun People's Hospital | Clinical practice | Diagnosis and treatment of hypertension |
|  | 2023.06 | “Chinese Elderly Hypertension Management Guidelines 2023” | Chinese Geriatrics Society Hypertension Branch, etc | Clinical guidelines | Management |
| The United States | 2017.09 | 《2017 Guideline for the Prevention, Detection, Evaluation, and Management of High Blood Pressure in Adults》 | American College of Cardiology, etc | Clinical guidelines | Diagnosis and treatment of hypertension |
|  | 2019.05 | 《Measurement of Blood Pressure in Humans A Scientific Statement From the American Heart Association》 | American Heart Association | Scientific statement | Blood pressure monitoring |
|  | 2021.06 | 《Management of Stage 1 Hypertension in Adults With a Low 10-Year Risk for Cardiovascular Disease: A Scientific Statement》 | American Heart Association | Scientific statement | Management |
|  | 2021.08 | 《Physical Activity as a Critical Component of First-Line Treatment for Elevated Blood Pressure or Cholesterol》 | American Heart Association | Scientific statement | Management |
|  | 2021.11 | 《Weight-Loss Strategies for Prevention and Treatment of Hypertension: A Scientific Statement From the American Heart Association》 | American Heart Association | Scientific statement | Management |
|  | 2022.01 | 《Medication Adherence and Blood Pressure Control: A Scientific Statement From the American Heart Association》 | American Heart Association | Scientific statement | Hypertension medication |
|  | 2022.02 | 《Hypertension in Pregnancy: Diagnosis, Blood Pressure Goals, and Pharmacotherapy: A Scientific Statement From the American Heart Association》 | American Heart Association | Scientific statement | Diagnosis and treatment of hypertension |
|  | 2022.07 | 《Ambulatory Blood Pressure Monitoring in Children and Adolescents: 2022 Update: A Scientific Statement From the American Heart Association》 | American Heart Association | Scientific statement | Blood pressure monitoring |
|  | 2023.10 | 《Implementation Strategies to Improve Blood Pressure Control in the United States: A Scientific Statement From the American Heart Association》 | American Heart Association, etc | Scientific statement | Management |
| International organization | 2020.05 | 《2020 ISH Global Hypertension Guidelines》 | International Society for Hypertension | Clinical guidelines | Management |
|  | 2021.08 | 《Guideline for the pharmacological treatment of hypertension in adults》 | World Health Organization | Clinical guidelines | Hypertension medication |
|  | 2023.09 | 《Global report on hypertension: The race against a silent killer》 | World Health Organization | Investigation report | Management |

Table 8 Summary of business needs for hypertension management

| Country | Business requirement dimension | Specific requirements | | |
| --- | --- | --- | --- | --- |
| China | Indicator measurement | Blood pressure measurement | Heart rate measurement | Weakness assessment |
|  |  | Psychological stress measurement | Blood oxygen measurement | BMI calculation |
|  |  | Cognitive function measurement | Sleep quality measurement |  |
|  | Indicator records | Blood pressure recording | Blood oxygen record | Sports records |
|  |  | Body posture information recording | Sleep recording | Adverse reaction records |
|  |  | Heart rate recording | Dietary records | Record of lifestyle habits |
|  |  | Medical history records | Medication records |  |
|  | Indicator trend tracking | Dynamic changes in blood pressure | Dynamic changes in heart rate | Dynamic display of electrocardiogram |
|  | Device Connection | Connecting a blood pressure monitor | Blood pressure meter calibration | Automatic data upload |
|  | Health intervention measures | Medication intervention | Exercise intervention | Prohibition intervention |
|  |  | Arrhythmia warning | Psychological intervention | Follow up of hypertensive patients |
|  |  | Dietary intervention | Smoking cessation intervention | Health Education |
|  | Personalized needs | Setting blood pressure control goals/treatment plans for different populations | Selection of blood pressure monitors for different populations | Guiding individualized treatment of hypertension |
|  |  | online pharmacy |  |  |
|  | Intelligent diagnosis of hypertension | Predicting the risk of hypertension | Classification of Hypertension Phenotypes | Screening of high-risk populations |
|  |  | Diagnosis of hypertension | Blood pressure grading | Interpretation of blood pressure reports |
|  |  | Risk assessment of cardiovascular and cerebrovascular diseases | Classification of causes of hypertension | Mental stress grading |
|  |  | Sleep disease risk prediction |  |  |
|  | Measurement/evaluation of therapeutic effects | Evaluate the effect of blood pressure reduction | Measurement accuracy |  |
|  | Doctor communication | Sharing dynamic blood pressure reports | Online expert consultation | Remote hypertensive consultation |
|  | Health support | Social support | Family support |  |
| The United States | Indicator measurement | Blood pressure measurement | Heart rate measurement | BMI calculation |
|  |  | Psychological stress measurement | Blood oxygen measurement | Sleep quality measurement |
|  | Indicator records | Blood pressure recording | Blood oxygen record | Sports records |
|  |  | Body posture information recording | Sleep recording | Adverse reaction records |
|  |  | Heart rate recording | Dietary records | Medical history records |
|  |  | Medication records |  |  |
|  | Indicator trend tracking | Dynamic changes in blood pressure | Dynamic changes in heart rate | Dynamic display of electrocardiogram |
|  | Device connection | Connecting a blood pressure monitor | Blood pressure meter calibration | Automatic data upload |
|  | Health intervention measures | Medication intervention | Dietary intervention | Smoking cessation intervention |
|  |  | Health Education | Exercise intervention | Prohibition intervention |
|  |  | Psychological intervention | Follow up of hypertensive patients |  |
|  | Personalized needs | Setting blood pressure control goals/treatment plans for different populations | Online pharmacy | Selection of blood pressure measurement methods for different populations |
|  |  | Selection of blood pressure monitors for different populations | Reward mechanism for promoting health |  |
|  | Intelligent diagnosis of hypertension | Diagnosis of hypertension | Blood pressure grading | Classification of Hypertension Phenotypes |
|  |  | Risk assessment of cardiovascular and cerebrovascular diseases | Classification of causes of hypertension | Screening of high-risk populations |
|  | Measurement/evaluation of therapeutic effects | Evaluate the effect of blood pressure reduction | Measurement accuracy |  |
|  | Doctor communication | Sharing dynamic blood pressure reports | Online expert consultation | Remote hypertensive consultation |
|  | Health support | Social support | Family support |  |
|  | Reliability of treatment plan | Standard treatment plan |  |  |
|  | Software performance | Software reliability | Software usability |  |

Table 9 Summary of functions of hypertension management app

| App feature dimensions | App specific features | Number of app |
| --- | --- | --- |
| Index measurement | Blood pressure measurement | 82 |
| Index measurement | Heart rate measurement | 89 |
| Index measurement | Blood oxygen measurement | 11 |
| Index measurement | Sleep measurement | 6 |
| Index measurement | BMI calculation | 10 |
| Index measurement | Temperature measurement | 1 |
| Index measurement | Psychological measurement | 1 |
| Indicator record | Blood pressure recording | 243 |
| Indicator record | Heart rate recording | 181 |
| Indicator record | Blood oxygen record | 30 |
| Indicator record | Sleep recording | 8 |
| Indicator record | Blood glucose recording | 36 |
| Indicator record | Blood lipid record | 8 |
| Indicator record | Weight Record | 9 |
| Indicator record | Temperature record | 1 |
| Indicator record | EHR management | 6 |
| Indicator trend tracking | Blood pressure trend | 187 |
| Indicator trend tracking | Heart rate trend | 111 |
| Indicator trend tracking | Blood glucose trend | 2 |
| Indicator trend tracking | Blood oxygen trend | 1 |
| Indicator trend tracking | Electrocardiogram | 7 |
| Health intervention measures | Bp measurement reminder | 52 |
| Health intervention measures | Hypertension knowledge base | 48 |
| Health intervention measures | Health message push | 24 |
| Health intervention measures | Medication management | 49 |
| Health intervention measures | Ischemic preconditioning training | 2 |
| Health intervention measures | Dietary intervention | 39 |
| Health intervention measures | Exercise intervention | 35 |
| Health intervention measures | Respiratory training | 3 |
| Intelligent diagnosis | Interpretation of Results | 58 |
| Intelligent diagnosis | Blood pressure classification | 76 |
| Intelligent diagnosis | Blood pressure warning | 28 |
| Intelligent diagnosis | Heart rate warning | 9 |
| Intelligent diagnosis | Psychological stress warning | 1 |
| Intelligent diagnosis | Online intelligent diagnosis | 12 |
| Doctor exchanges | Doctor-patient communication | 25 |
| Doctor exchanges | Expert consultation | 21 |
| Doctor exchanges | Share results | 120 |
| Device connect | Connecting a bp monitor | 73 |
| Device connect | Connect the smart bracelet | 27 |
| Device connect | Automatic data upload | 57 |
| Device connect | Data backup | 8 |
| Device connect | Bp meter calibration | 2 |
| Health support | Online community | 10 |
| Health support | Family bp management | 70 |
| Personalized features | Provide optional languages | 68 |
| Personalized features | Online shopping mall | 8 |
| Personalized features | Task check-in | 9 |
| Personalized features | Points mall | 5 |
| Personalized features | Memorandum | 32 |

Table 10 The proportion of user reviews corresponding to the theme of the influencing factors of user satisfaction in China and the United States

| Country | Theme categories | Theme (influencing factors) | Keywords (Chinese/English) | Number of reviews (%) |
| --- | --- | --- | --- | --- |
| China | Software availability | Reliability | Health, quality, comprehensiveness, support, decent, professional, comprehensive | 2,165(13.07%) |
|  |  | Compatibility | Version, download, try, blood pressure monitor, Apple, connection, platform | 878(5.30%) |
|  | Software usability | Convenience | Not bad, convenient, easy to use, practical, quite useful, recommended, easy to operate | 6,863(41.44%) |
|  |  | Page design | Update, special, interface, clear, design, components, good-looking | 471(2.84%) |
|  | Software monitoring function | Heart rate monitoring | Heart rate, detection, numerical value, body, monitoring, watch, indicators | 597(3.60%) |
|  |  | Blood pressure tracking | Blood pressure, function, recording, tools, data, sharing, tables | 502(3.03%) |
|  | Software monitoring effect | Measurement accuracy | Measurement, accuracy, heart rate, accuracy, testing, data, inaccuracy | 2,091(12.63%) |
|  |  | Real time monitoring | Trial, view, status, anytime, anywhere, monitoring, patient, daily | 299(1.81%) |
|  | User attitude | Positive evaluation | Very good, like, recommend, everyday, awesome, useful, expected | 1,850(11.17%) |
|  | Data management | Data privacy | Account, Personal, Information, Security, Management, Privacy, Licensing | 192(1.17%) |
|  | Software cost | Cost | Fee, subscription, free, payment, upgrade, refund, payment | 653(3.94%) |
| The United States | Software availability | Reliability | quality, bad, screen, complete, fake, uninstalling, bug | 3,793(3.79%) |
|  |  | Compatibility | version, iphone, upgrade, fine, android, fail, reinstall | 2,586(2.58%) |
|  | Software usability | Convenience | easy, love, simple, record, report, recommend, worth | 34,443(34.40%) |
|  |  | Page design | user, wonderful, friendly, interface, experience, unit, type | 2,390(2.39%) |
|  |  | Advertising distribution | advertisement, download, watch, garbage, click, poor, difficult | 1,599(1.60%) |
|  | Software monitoring function | Blood pressure tracking | blood, pressure, monitor, track, check, record, measure | 17,285(17.26%) |
|  |  | Heart rate monitoring | heart, rate, pulse, check, measure, test, monitor | 7,065(7.06%) |
|  | Software monitoring effect | Effect of blood pressure management | good, help, care, body, hypertension, maintain, condition | 11,881(11.86%) |
|  |  | Measurement accuracy | accurate, cuff, feel, offer, result, manual, actual | 1,199(1.20%) |
|  | Data management | Data synchronization | time, phone, update, synchronization, connect, omron, data | 12,837(12.82%) |
|  |  | Data sharing | data, email, export, send, share, access, require | 4,465(4.46%) |
|  | Software cost | Cost | cost, fee, free, money, afford, pay, count | 582(0.58%) |

Table 11 The frequency and proportion of user satisfaction evaluation indicators in China and the United States

| Country | Criterion layer | Indicator layer | Ratio of indicator frequency |
| --- | --- | --- | --- |
| China | Software availability (18.37%) | Reliability | 13.07% |
|  |  | Compatibility | 5.30% |
|  | Software usability (44.28%) | Convenience | 41.44% |
|  |  | Page design | 2.84% |
|  | Software monitoring function (6.63%) | Heart rate monitoring | 3.60% |
|  |  | Blood pressure tracking | 3.03% |
|  | Software monitoring effect (14.44%) | Measurement accuracy | 12.63% |
|  |  | Real time monitoring | 1.81% |
|  | Data Management (1.17%) | Data privacy | 1.17% |
|  | Software cost (3.94%) | Cost | 3.94% |
| The United States | Software availability (6.37%) | Reliability | 3.79% |
|  |  | Compatibility | 2.58% |
|  | Software usability (38.39%) | Convenience | 34.40% |
|  |  | Page design | 2.39% |
|  |  | Advertising distribution | 1.60% |
|  | Software monitoring function (24.32%) | Blood pressure tracking | 17.26% |
|  |  | Heart rate monitoring | 7.06% |
|  | Software monitoring effect (13.06%) | Effect of blood pressure management | 11.86% |
|  |  | Measurement accuracy | 1.20% |
|  | Data management (17.28%) | Data synchronization | 12.82% |
|  |  | Data sharing | 4.46% |
|  | Software cost (0.58%) | Cost | 0.58% |

Table 12 The importance scale of user satisfaction evaluation indicator systems in China and the United States

| Importance classification | Scale value | Difference in the proportion of China indicator frequency | Difference in the proportion of US indicator frequency |
| --- | --- | --- | --- |
| Equally important | 1 | [0.00%, 4.79%] | [0.00%, 4.20%] |
| Slightly important | 3 | (9.58%, 14.37%] | (8.40%, 12.60%] |
| More important | 5 | (19.16%, 23.95%] | (16.80%, 21.00%] |
| Very important | 7 | (28.74%, 33.53%] | (25.20%, 29.40%] |
| Absolutely important | 9 | (38.32%, +∞) | (33.60%, +∞) |
| Other | 2, 4, 6, 8 | Between the proportion differences of different importance levels | Between the proportion differences of different importance levels |

Table 13 The comprehensive weight of the US user satisfaction evaluation indicator system

| Target layer | Criterion layer | Criteria layer weights | Indicator layer | Indicator layer weight | Comprehensive weight |
| --- | --- | --- | --- | --- | --- |
| User satisfaction | Software availability | 4.80% | Reliability | 50.00% | 2.40% |
|  |  |  | Compatibility | 50.00% | 2.40% |
|  | Software usability | 52.49% | Convenience | 80.00% | 41.99% |
|  |  |  | Page design | 10.00% | 5.25% |
|  |  |  | Advertising distribution | 10.00% | 5.25% |
|  | Software monitoring function | 19.94% | Blood pressure tracking | 75.00% | 14.95% |
|  |  |  | Heart rate monitoring | 25.00% | 4.98% |
|  | Software monitoring effect | 7.62% | Effect of blood pressure management | 75.00% | 5.72% |
|  |  |  | Measurement accuracy | 25.00% | 1.91% |
|  | Data management | 11.86% | Data synchronization | 66.67% | 7.91% |
|  |  |  | Data sharing | 33.33% | 3.95% |
|  | Software cost | 3.29% | Cost | 100.00% | 3.29% |

Table 14 Combination consistency test and overall consistency test of the US user satisfaction evaluation indicator system

| Judgment matrix hierarchy | | Weight | CI | RI | CR^(N)^ | CR* |
| --- | --- | --- | --- | --- | --- | --- |
| Criterion layer | Target layer-criterion layer | 1 | 0.0374 | 1.26 | 0.0297<0.1 | 0.0297<0.1 |
| Indicator layer | Software availability-indicator layer | 0.0480 | 0 | 0 | 0<0.1 |  |
|  | Software usability-indicator layer | 0.5249 | 0 | 0.52 |  |  |
|  | Software monitoring function-indicator layer | 0.1994 | 0 | 0 |  |  |
|  | Software monitoring effectiveness-indicator layer | 0.0762 | 0 | 0 |  |  |
|  | Data management-indicator layer | 0.1186 | 0 | 0 |  |  |
|  | Software cost-Indicator layer | 0.0329 | 0 | 0 |  |  |

CR^(N)^ represents the combination consistency coefficient of the Nth layer, CR* represents the overall consistency coefficient of the evaluation indicator system.

Table 15 The comprehensive weights of China’s user satisfaction evaluation indicator system

| Target layer | Criterion layer | Criteria layer weights | Indicator layer | Indicator layer weight | Comprehensive weight |
| --- | --- | --- | --- | --- | --- |
| User satisfaction | Software availability | 14.67% | Reliability | 66.67% | 9.78% |
|  |  |  | Compatibility | 33.33% | 4.88% |
|  | Software usability | 58.29% | Convenience | 90.00% | 52.46% |
|  |  |  | Page design | 10.00% | 5.83% |
|  | Software monitoring function | 6.07% | Heart rate monitoring | 50.00% | 3.04% |
|  |  |  | Blood pressure tracking | 50.00% | 3.04% |
|  | Software monitoring effect | 12.00% | Measurement accuracy | 75.00% | 9.00% |
|  |  |  | Real time monitoring | 25.00% | 3.00% |
|  | Data management | 4.24% | Data privacy | 100.00% | 4.24% |
|  | Software cost | 4.73% | Cost | 100.00% | 4.73% |

Table 16 Combination consistency test and overall consistency test of the Chinese user satisfaction evaluation indicator system

| Judgment matrix hierarchy | | Weight | CI | RI | CR^(N)^ | CR* |
| --- | --- | --- | --- | --- | --- | --- |
| Criterion layer | Target layer-criterion layer | 1 | 0.0338 | 1.26 | 0.0268<0.1 | 0.0268<0.1 |
| Indicator layer | Software availability-indicator layer | 0.1467 | 0 | 0 | 0<0.1 |  |
|  | Software usability-indicator layer | 0.5829 | 0 | 0 |  |  |
|  | Software monitoring function-indicator layer | 0.0607 | 0 | 0 |  |  |
|  | Software monitoring effectiveness-indicator layer | 0.1200 | 0 | 0 |  |  |
|  | Data management-indicator layer | 0.0424 | 0 | 0 |  |  |
|  | Software cost-indicator layer | 0.0473 | 0 | 0 |  |  |

CR^(N)^ represents the combination consistency coefficient of the Nth layer, CR* represents the overall consistency coefficient of the evaluation indicator system.

**6 Summary of Figure**


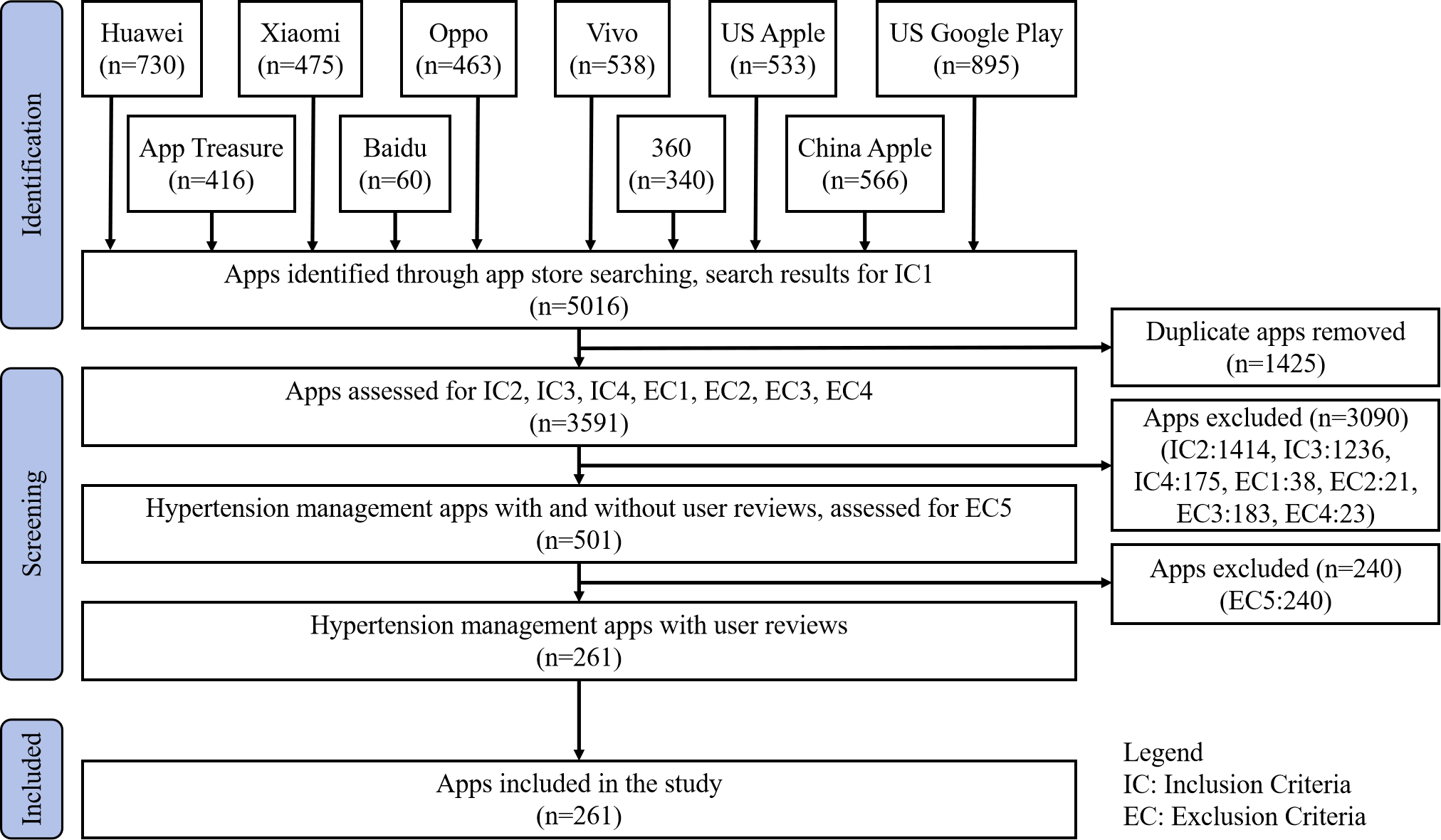


Figure 1 Flowchart of the hypertension mobile health app screening process


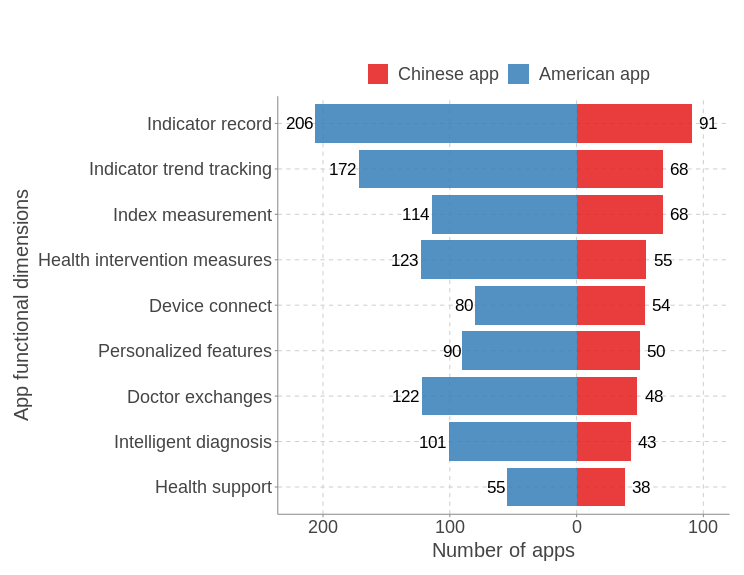


Figure 2 Dimensional distribution of functional characteristics of hypertension management apps in the United States and China


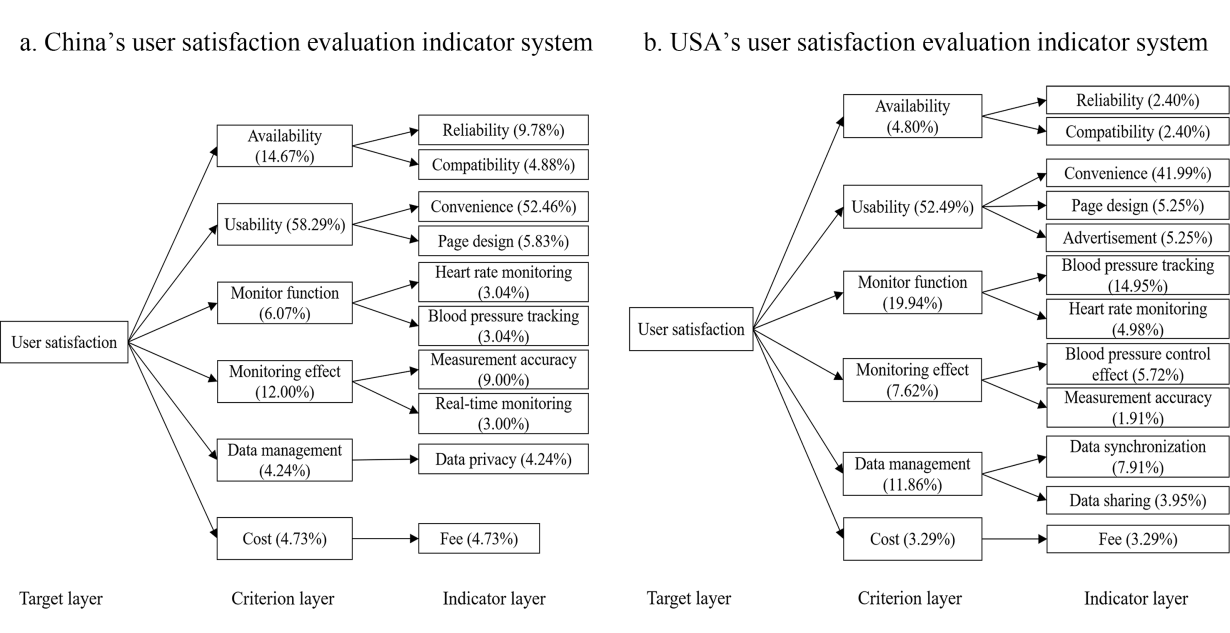


Figure 3 Framework of user satisfaction evaluation indicator system in the United States and China


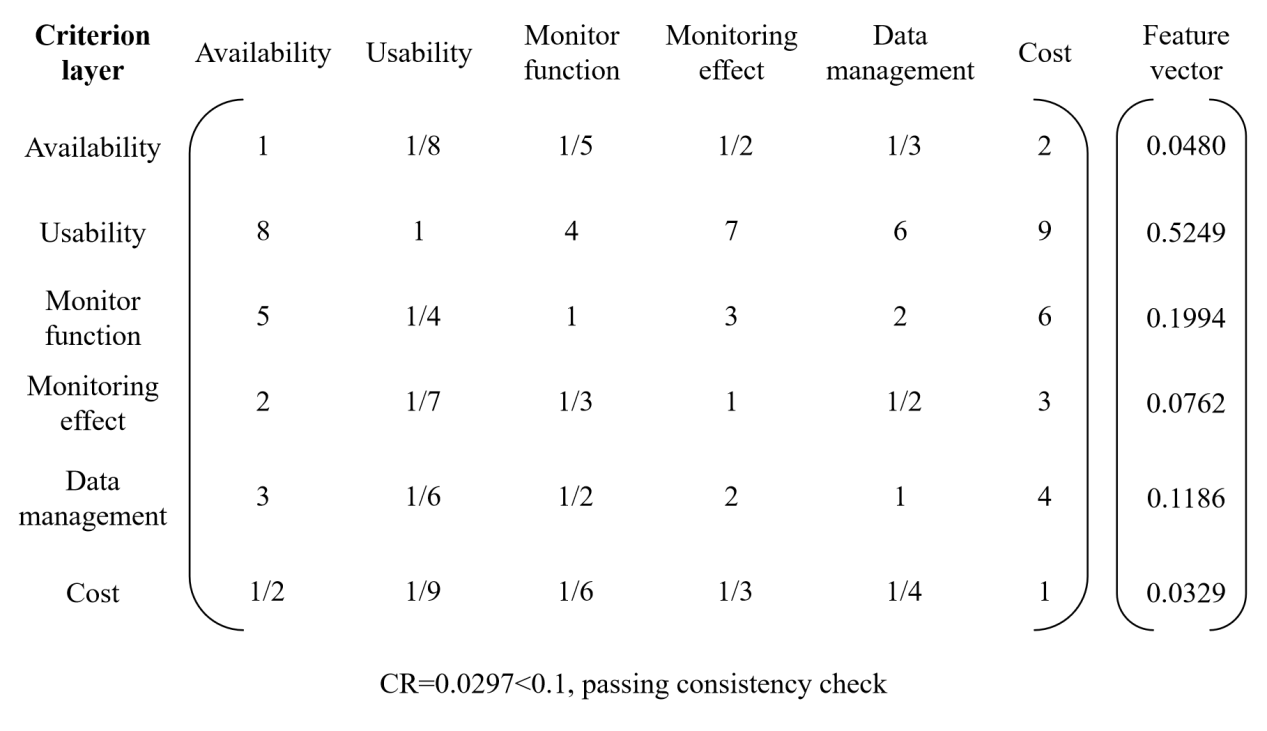


Figure 4 Criterion layer judgment matrix of the US user satisfaction evaluation indicator system


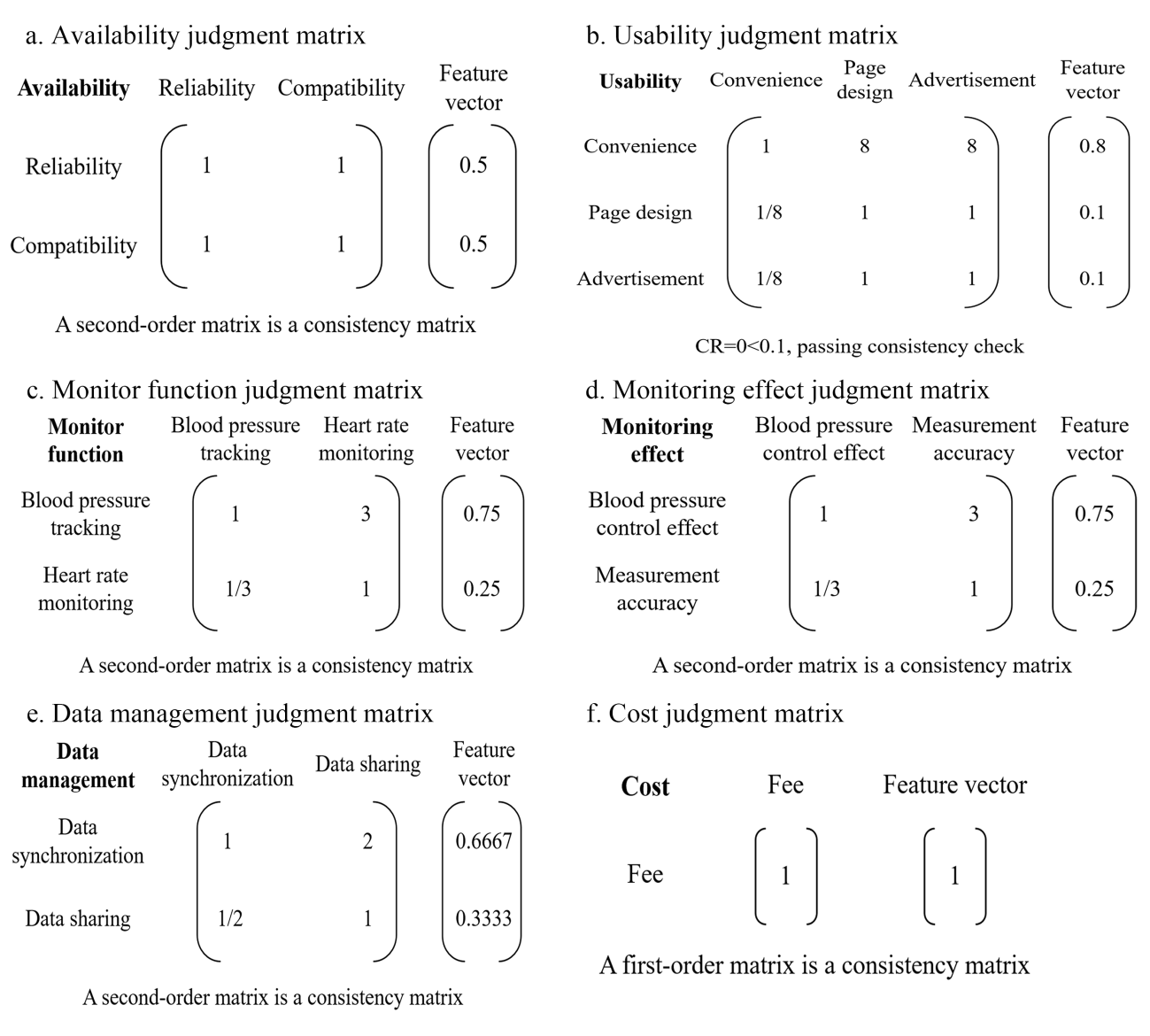


Figure 5 The indicator layer judgment matrix of the US user satisfaction evaluation indicator system


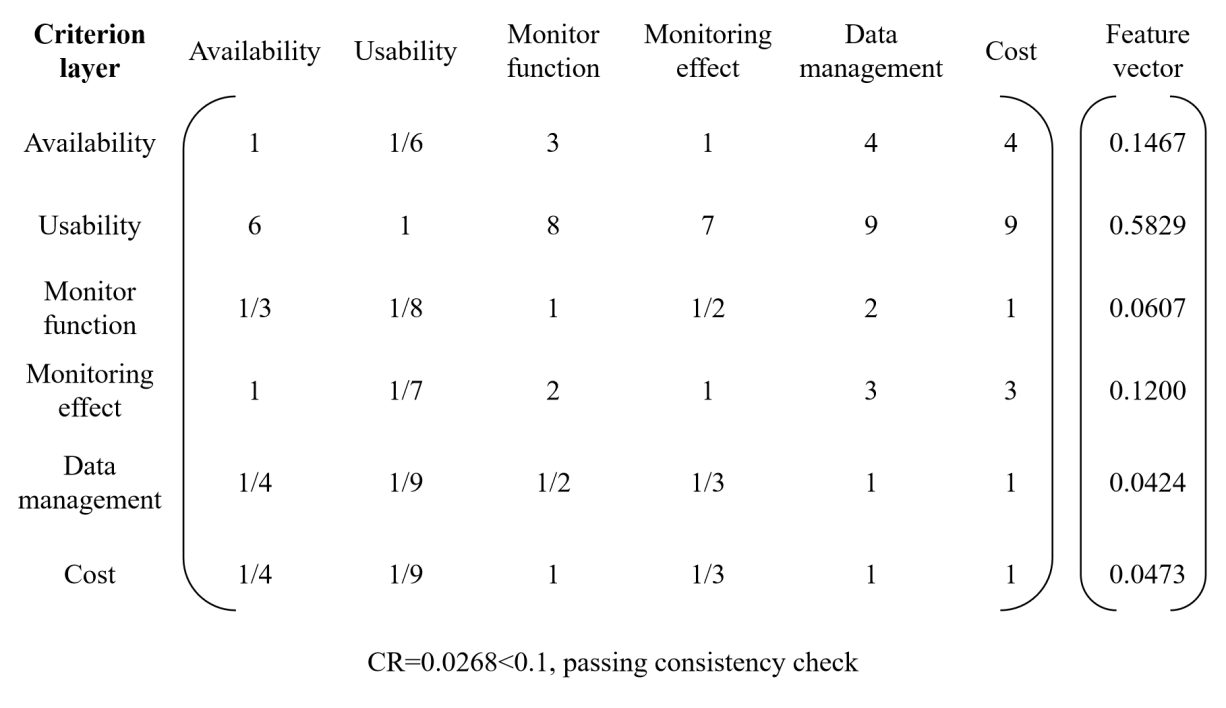


Figure 6 Criterion layer judgment matrix of China’s user satisfaction evaluation indicator system


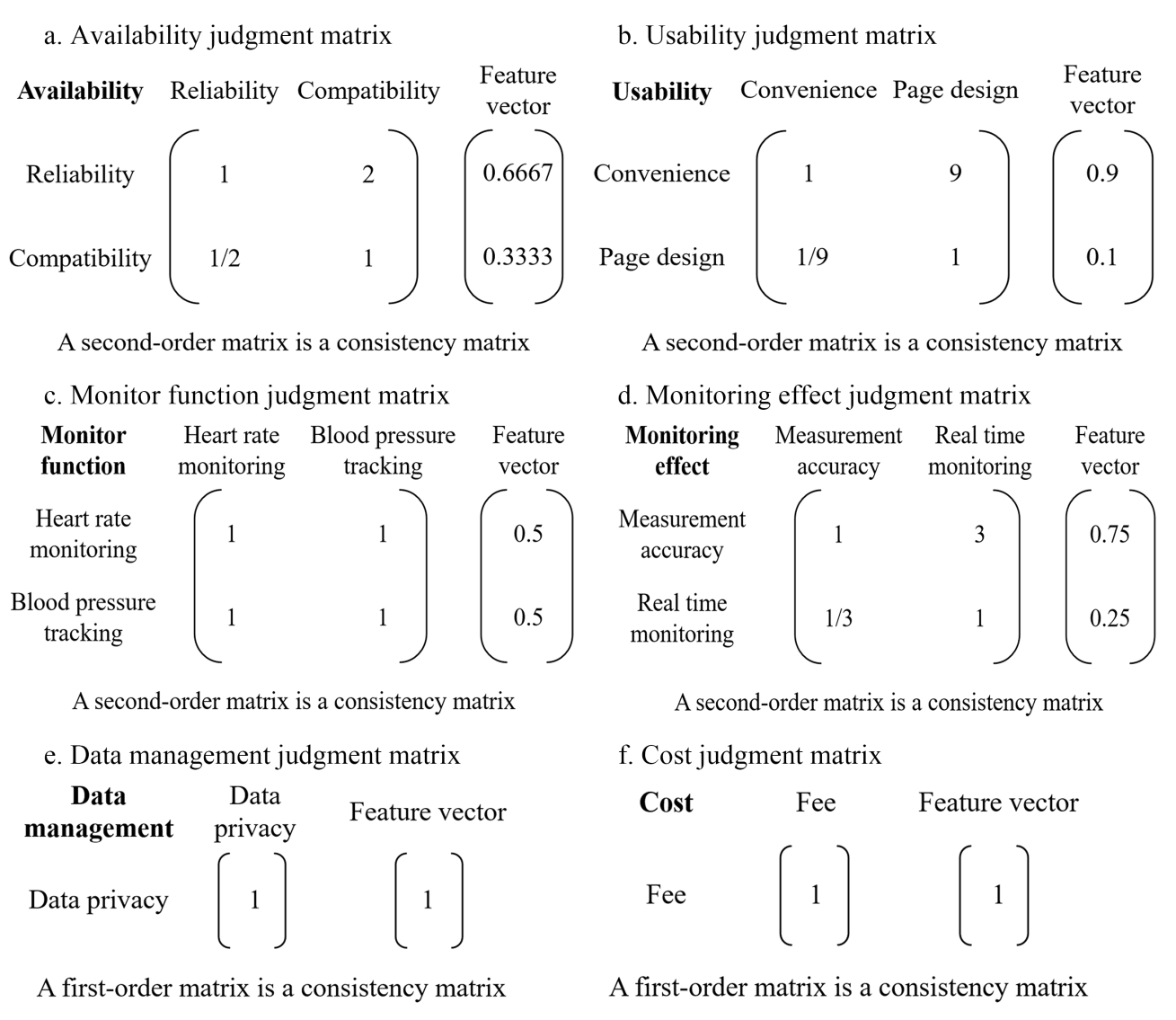


Figure 7 The indicator layer judgment matrix of China’s user satisfaction evaluation indicator system
